# Humanized tau antibodies promote tau uptake by human microglia without any increase of inflammation

**DOI:** 10.1186/s40478-020-00948-z

**Published:** 2020-05-29

**Authors:** Monika Zilkova, Anna Nolle, Branislav Kovacech, Eva Kontsekova, Petronela Weisova, Peter Filipcik, Rostislav Skrabana, Michal Prcina, Tomas Hromadka, Ondrej Cehlar, Gabriela Paulikova Rolkova, Denisa Maderova, Michal Novak, Norbert Zilka, Jeroen J. M. Hoozemans

**Affiliations:** 1 Axon Neuroscience R&D Services SE, Dvorakovo nabrezie, 10 Bratislava, Slovak Republic; 2 Amsterdam UMC, Vrije Universiteit Amsterdam, Department of Pathology, Amsterdam Neuroscience, De Boelelaan, 1117 Amsterdam, The Netherlands; 3 Axon Neuroscience SE, Arch. Makariou & Kalogreon 4, Larnaca, Cyprus

**Keywords:** Tau immunotherapy, Humanized antibody, Human microglia, Tau uptake

## Abstract

Immunotherapies targeting pathological tau have recently emerged as a promising approach for treatment of neurodegenerative disorders. We have previously showed that the mouse antibody DC8E8 discriminates between healthy and pathological tau, reduces tau pathology in murine tauopathy models and inhibits neuronal internalization of AD tau species in vitro.

Here we show, that DC8E8 and antibodies elicited against the first-in-man tau vaccine, AADvac1, which is based on the DC8E8 epitope peptide, both promote uptake of pathological tau by mouse primary microglia. IgG1 and IgG4 isotypes of AX004, the humanized versions of DC8E8, accelerate tau uptake by human primary microglia isolated from post-mortem aged and diseased brains. This promoting activity requires the presence of the Fc-domain of the antibodies.

The IgG1 isotype of AX004 showed greater ability to promote tau uptake compared to the IgG4 isotype, while none of the antibody-tau complexes provoked increased pro-inflammatory activity of microglia. Our data suggest that IgG1 has better suitability for therapeutic development.

## Introduction

Several tau immunotherapeutic approaches are currently being developed as treatment for tau pathology in Alzheimer’s disease [44]. Anti-tau antibodies are believed to be a promising tool to block uptake [51] and spreading of tau seeds by neurons and to stimulate the removal of tau seeds by microglia [recently reviewed in 36].

The antibody-mediated uptake and degradation of pathological extracellular tau by microglia is an extensively discussed issue of tau immunotherapy due to the unclear contribution of microglia to the pathophysiology of neurodegenerative disorders [4, 28, 46, 49, 52]. Microglia, resident immune cells of the brain, have important physiological functions in maintaining tissue homeostasis. They respond to changes in the microenvironment by diverse range of phenotypes with both neuroinflammatory and neuroprotective properties, which makes their role in neurodegeneration less transparent [20, 49, 50].

The selection of the isotype of a therapeutic antibody is an important aspect of its development, since antibody isotypes differently influence the receptor-mediated uptake by immune cells, induction of cytokines and engagement of various arms of the immune system [6, 12]. Choice of the optimal IgG isotype for a therapeutic anti-tau antibody depends both on efficacy and safety. The human IgG1 isotype exhibits the full effector function, while for example the IgG4 isotype has lower affinities to Fc-γ receptors and does not activate the complement cascade [12]. Some successful preclinical studies performed on mouse model systems used either IgG1, IgG2a or IgG2b isotypes [9, 19, 27, 53]. However, only a limited number of studies directly compared the potency of different anti-tau antibody isotypes.

Advances in the cell culture methods allowed cultivation of human primary microglia from the brains of diseased patients [35]. These cells exhibit chronic activation and pro-inflammatory phenotype reflecting the CNS microenvironment of aged and diseased brains, and differ from aged murine microglia models [16, 18, 45]. Up to now, however, no human primary microglia were used for preclinical testing of anti-tau antibodies.

In this work we compared IgG1 and IgG4 isotypes of a novel anti-tau therapeutic antibody AX004, a humanized version of the mouse monoclonal antibody DC8E8 [23], in their ability to potentiate phagocytosis of oligomeric extracellular tau by human microglia. DC8E8 promotes tau uptake by mouse neonatal microglia. Importantly, mouse serum antibodies elicited by a vaccine based on the DC8E8 epitope peptide, AADvac1, showed similar promoting activity suggesting that they share a common mechanism of action with DC8E8. In order to translate these results to the diseased human brain, we used primary human microglia isolated from post-mortem brains of patients with and without neurodegenerative disorders. Both IgG1 and IgG4 isotypes of AX004 stimulated uptake of abnormal tau proteins by human adult primary microglia. Our results showed that humanized antibody-tau complexes do not provoke higher inflammatory response in comparison to tau alone. The higher activity of the IgG1 isotype in enhancing tau internalization compared to IgG4 suggests that IgG1 will be more effective in the removal of pathological tau proteins from the human brains and should be more suitable for therapeutic development.

## Material and methods

### Antibodies

Monoclonal anti-tau antibodies AX004/IgG1 and AX004/IgG4, humanized version of anti-tau monoclonal antibody DC8E8 [23], and their Fab fragment were produced in Expi-CHO cells (Thermo Fisher Cat. No. A29133). The expression of antibodies was achieved with the MaxCyte STX Flow ElectroporationTM platform (Gaithersburg, USA) after the transient transfection of plasmids bearing the sequence coding for heavy and light chains of AX004. 8e7 cells were used for electroporation in a small scale. Cells were incubated in serum free, protein and antibiotic free medium in Caron CO2 (USA) incubator at 35 °C and 5% of CO2. Cultures were placed on advanced Dura-Shaker (Triad scientific) for extreme environments and orbitally shaken at 120 rpm. 24 h after the electroporation the cells were supplemented with the sodium butyrate and feed and cultured further for 14 days. Purification of antibodies was performed by affinity chromatography on Protein A, followed by cation-exchange chromatography and buffer exchange to PBS. The activity of purified antibodies was verified by ELISA, Western blot and immunohistochemistry. The tau binding properties of the Fab fragment were verified by surface plasmon resonance (SPR) on Biacore3000 and showed comparable binding affinity to the full antibody AX004.

Anti-human antibodies CD11b-PE-Vio770, CD64-Vio615, CD32-PE, CD16-VioBright and controls: REA control-Vio770, REA control-Vio615, REA control –PE, REA control-VioBright (MACS Miltenyi Biotec), anti-Iba1 (WAKO) were used for human microglia.

Monoclonal mouse antibodies DC8E8 and DC25 [23], DC51 [32], DC190 (mapping tau epitope 368–376, Axon Neuroscience SE), rabbit anti-Iba1 (WAKO), blocking antibodies anti-CD16 + CD32 (Abcam) and anti-CD64 (SantaCruz Biotechnologies), rat anti-mouse CD11b Pacific Blue (Biorad), mouse IgG2b isotype control Pacific Blue (Biorad), PE rat anti-mouse CD16/CD32 (BD PharmingenTM), PE mouse IgG2b isotype control (BD PharmingenTM), mouse FcgRIA/CD64a (A594) (R&D systems), and mouse IgG2a isotype control (A594) were used for tau analysis and for mouse microglia.

The Fab fragment of mouse monoclonal antibody DC8E8 was prepared by partial digestion of the full-length antibody with papain as described previously [8, 23]. Activities of Fab fragments were verified by SPR.

### Isolation of primary human microglia from brain tissue

Primary microglia were isolated from human brain tissue by density gradient centrifugation as previously described by de Groot et al. [11]. Corpus callosum white matter was acquired from rapid autopsy according to the standard protocols of the Netherlands Brain Bank (Amsterdam, The Netherlands). Brain donors signed informed consent for autopsy and the use of tissue and medical records for research purposes (see Table 1 for used donors). This study was approved by the ethical committee of the VUmc. After dissection the tissue was collected in collection medium (Dulbecco’s modified Eagle medium [DMEM] and Ham F10 1:1 supplemented with 50 μg/ml gentamycin). The tissue was washed with phosphate-buffered saline (PBS) and homogenized with a scalpel in a trypsin-EDTA solution (Thermofisher) and incubated at 37 °C for 20 min. The homogenized tissue was washed with culture medium (DMEM and Ham F10 1:1 supplemented with 10% heat inactivated fetal bovine serum (FBS [Hyclone, Thermo Fisher Scientific], a mixture of 100 IU/mL penicillin and 100 IU/mL streptomycin [Gibco], and 0.5% L-glutamine), collected via centrifugation at 1785 RCF, suspended again in culture medium, filtered through a 100 μm-pore filter, and pelleted again by centrifugation at 1785 RCF. It was then resuspended in a solution of myelin gradient buffer (3.56 g/L disodium phosphate and 0.78 g/L monosodium phosphate buffer with 140 mM NaCl, 5.4 mM KCl, 11 mM glucose, and 0.2% BSA, pH 7.4), Percoll, and 1.5 M aqueous NaCl. Density gradient centrifugation at 2474 RCF was performed to procure a microglia-enriched cell pellet, which was washed once with culture medium to remove any remaining Percoll beads, then resuspended in a hypotonic shock solution (0.15 M NH_4_Cl and 1 mM KHCO_3_ supplemented with 0.75% BSA) and incubated at 4 °C for 15 min to lyse remaining erythrocytes contaminating the pellet. The microglia were washed once more with culture medium, pelleted via centrifugation at 723 RCF, resuspended in culture medium, seeded in 24-well uncoated, electrostatically-treated culture plates (Corning Costar), and incubated at 37° with 5% CO_2_. 24 h after isolation, the microglia were treated with 25 μg/mL granulocyte macrophage colony stimulating factor (recombinant human GM-CSF, Immunotools) to allow for better adherence and proliferation. Microglia were utilized in experiments 8–14 days post-isolation.

**Table 1:** Brain donors used for isolation of human primary microglia from corpus callosum white matter brain tissue

Donor number	Age of death (years)	Gender	PMD (hrs:min)	Clin Diag
1	21	m	08:55	control
2	29	f	07:00	control
3	71	m	05:40	control
4	82	f	06:48	control
5	82	f	06:20	control
6	70	f	07:00	control
7	89	m	05:55	AD
8	80	f	07:05	AD
9	86	m	03:44	AD
10	74	m	05:15	PD
11	88	m	06:45	PD
12	61	f	10:50	FTD
13	71	m	04:55	DLB
14	84	f	08:40	PSP
15	86	f	05:15	PSP
16	75	m	09:05	MS
17	74	f	05:05	MSA
18	70	f	06:45	MSA

*M* Male, *f* Female, *PMD* Post-mortem delay; control, non-neurological control (absence of neuropathological conditions); *AD* Alzheimer’s disease, *FTD* Frontotemporal dementia, *DLB* Dementia with Lewy bodies, *PSP* Progressive supranuclear palsy, *MS* Multiple sclerosis, *MSA* Multiple system atrophy.

### Primary mouse microglial culture

Cerebral cortices of 1-day old newborn C57BL/6NCRL mice (Charles River) were dissected by cervical dislocation, stripped of their meninges, and mechanically dissociated by repeated pipetting followed by passing through a nylon mesh. Cells were plated in 12-well plates pre-coated with poly-L-lysine (10 mg/ml) and cultivated in DMEM containing 10% FCS and 2 mM L-glutamine (all from Life Technologies Invitrogen, Carlsbad, California, United States) at 37 °C, 5% CO2 in a water-saturated atmosphere. The medium was changed twice a week. Mixed glial cultures reached confluence after 8 to 10 days in vitro. Confluent mixed glial cultures were subjected to mild trypsinization (0.06% trypsin-EDTA). This resulted in the detachment of an intact layer of cells containing astrocytes, leaving undisturbed a population of firmly attached cells [41]. Pure mouse microglia cells were re-plated into 12-well plate in a plating density 3 × 10^5^ cells/well, maintained in astrocyte-conditioned medium and were used for experiments after 24–48 h in culture. The purity of microglial cell cultures isolated by this procedure was routinely around 95% (CD11b staining).

All experiments on animals were carried out according to institutional animal care guidelines conforming to international standards and were approved by State Veterinary and Food Committee of Slovak Republic (Ro-4429/16-221b, Ro-2707/18–221/3) and by the Ethics Committee of the Institute of Neuroimmunology, Slovak Academy of Science, Bratislava.

### Purification of recombinant truncated tau protein and its oligomerization

Human truncated tau151–391/4R (numbering according to the longest human tau isoform Tau40) was expressed in *Escherichia coli* strain BL21(DE3) (Sigma-Aldrich, St. Louise, Missouri, United States) from a pET-17 expression vector and purified from bacterial lysates as described previously [10], except the size-exclusion chromatography was performed in PBS-argon (137 mM NaCl, 2.7 mM KCl, 10 mM Na2HPO4, 2 mM KH2PO4, pH 7.4) (AppliChem GmbH, Darmstadt, Germany). To avoid bacterial macromolecular contamination, tau protein was further immunoaffinity purified using the DC25 mAb column [24]. Purified tau protein was concentrated on a cation-exchange HiTrap SP Sepharose HP column and stored in PBS saturated with argon in working aliquots at − 70 °C [25]. The purity of tau protein was subsequently verified by gradient SDS gel electrophoresis (5 to 20% gel), Coomassie blue staining and Western blot analysis with DC25 antibody (AXON Neuroscience SE, Larnaca, Cyprus), which recognizes residues 347–353 of the longest human tau isoform Tau40.

In vitro oligomerization of recombinant truncated tau protein tau 151–391/4R was carried out at a concentration of 240 μM in PBS (137 mM NaCl, 2.7 mM KCl, 10 mM Na2HPO4, 2 mM KH2PO4, pH 7.4) using 60 μM heparin (Sigma-Aldrich, St. Louis, Missouri, United States) as an inducer [33]. The reaction was performed for 5 days at 37 °C. After incubation, tau oligomers were collected by ultracentrifugation at 100,000×g for 1 h at room temperature and the pellet was re-suspended in PBS and sonicated for 5 s at 20% power output using an MS72 probe of a Bandelin Sonopuls Sonifier (Bandelin, Berlin, Germany) and stored at − 70 °C. The oligomerization of the tau protein was confirmed by SDS gel electrophoresis, quantitative thioflavin T (ThT) fluorescence spectroscopy with excitation at 450 nm and emission at 510 nm, light-scattering measurements and infrared absorption spectroscopy.

Fluorescently tagged tau protein was prepared by labelling with Alexa Fluor 488 (Invitrogen, Carlsbad, California, USA) according to the manufacturer’s recommendations.

### Light scattering measurements

Ten microliter of oligomerized tau151–391/4R sample was centrifuged at 5000×g for 5 min at 25 °C, transferred into a 4 μl disposable cuvette (Wyatt Technology) and measured in a Dynapro NanoStar instrument controlled by Dynamics software v. 7.8.0.26 (Wyatt Technology). Measurements were performed in a 10 s acquisition time averaged 10-times. Data from 10 individual measurements of dynamic light scattering (DLS) per sample were evaluated by the Dynamics software version 7.8.1.3. To cull the acquisitions influenced by dust or irregular particles, an automatic filtering of autocorrelation functions was applied with an individual limits for baseline threshold and maximal allowed sum-of-squares (SOS) error for cumulants fit. After filtering, at least 65% of original data remained for analysis. To determine the size distribution of protein preparations, DLS autocorrelation data were subjected to a regularization analysis by Dynals algorithm. Final graphs were prepared in Prism 6 software (GraphPad).

### Infrared spectroscopy

Infrared spectra were collected on ThermoScientific Nicolet iS50R Research FTIR Spectrometer equipped with a DTGS detector (Thermo Fisher Scientific). The instrument and sampling accessory were continuously purged with air free from water vapor and CO_2_. One μl of tau151–391/4R monomer or tau151–391/4R oligomers pellet suspension in PBS was loaded into a ConcentratIR2 Multiple Reflection ATR sampling plate (Harrick Scientific Products) adopting a silicon element with a nominal incident angle of 30° and eleven reflections. The sampling plate was sealed and the sample drop dried under flow of dry air. After vanishing of liquid water absorption bands, the flow of dry air was stopped and 32 scans were collected at 4 cm^− 1^ resolution within a spectral range of 650–4000 cm^− 1^ wavenumbers. Spectra were collected using zero-filling factor 2, Happ-Genzel apodization, Mertz phase correction, aperture 160, sample gain 4, optical velocity 0.4747 cm.s^− 1^. Reference spectra were recorded under identical conditions with empty ATR sampling plate and were subtracted from the protein-sample spectra. Spectra were further baseline-corrected and processed with ATR advanced correction tool as implemented in OMNIC software v. 9 (Thermo Fisher Scientific).

### Animal vaccination and affinity purification of anti-tau antibodies from mouse sera

Female C57BL/6NCRL mice were immunized with three doses of the AADvac1 vaccine in 1-week intervals as described previously [22]. Four weeks after the last dose the animals were bled, the collected blood (pooled from 35 animals) was let to clot for 2 h at room temperature and then incubated overnight at + 2 to + 8 °C. Serum was separated by centrifugation at 2000 g for 10 min.

For affinity purification of anti-tau antibodies, recombinant truncated tau protein (tau151–391/4R) was coupled to superparamagnetic Dynabeads M280 Tosylactivated (ThermoFisher Scientific) according to manufacturer’s instructions. The prepared tau-beads were incubated with mouse serum 6-fold diluted with PBS supplemented with Tween 20 and Complete® protease inhibitors (Roche) at + 2 to + 8 °C for 16 h with head-over-tail rotation. The bound antibodies were eluted with 0.2 M glycine pH 2.7 and immediately neutralized with 1 M Tris-HCl pH 8.0.

Control antibodies were isolated from a pool of 10 non-immunized mice by using Dynabeads Protein G (GE Healthcare) and final concentration was determined by absorption spectroscopy.

Quality of AADvac1-induced tau-specific antibodies was analysed by SDS-polyacrylamide electrophoresis: 1 μg of AADvac1-induced tau-specific antibodies, control sera and DC8E8 were loaded onto 12% SDS polyacrylamide gel and electrophoresed in a Tris-glycine-SDS buffer system and stained with 0.05% Coomassie Blue R250 (Sigma Aldrich) in 10% acetic acid, 40% methanol in H_2_O.

The binding capacity of AADvac1-induced tau-specific antibodies to oligomerized fluorescently tagged tau protein with Alexa Fluor 488 was detected by sandwich enzyme-linked immunosorbent assay (sandwich ELISA).

### Enzyme-linked immunosorbent assay (ELISA)

The recombinant tau151–391/4R protein or oligomerized fluorescently tagged tau151–391/4R protein with Alexa Fluor 488 were immobilized on ELISA plates (Nunc, MediSorp) at 5 μg/ml in PBS, 50 μl/well, and incubated overnight at 37 °C. After blocking with PBS-0.05% Tween 20 (1 h at 20–25 °C), the plates were incubated with 50 μl/well of three-fold serial antibody dilutions (concentration range of 10,000 ng/ml – 0.05 ng/ml) in blocking buffer (PBS, 0.05% Tween 20) for 1 h at 37 °C. After incubation and washing, peroxidase-conjugated secondary antibody (for mouse antibodies: anti-mouse Ig, Dako; for humanized antibodies: anti-human Ig, Pierce, ThermoScientific) was diluted 1:4000 in PBS-Tween buffer and applied to the wells (50 μl/well) for 1 h at 37 °C. After washing, the reaction was developed for 20 min with TMB one solution (Kem En Tek Diagnostics, 50 μl/well) as a peroxidase substrate and stopped with 50 μl of 0.25 M H_2_SO_4_. Absorbance was measured at 450 nm using a Multiscan MCC/340 ELISA reader (Labsystems). Readouts with an absorbance value of at least twice the value of the negative controls (PBS) were considered positive.

### Immunohistochemistry

Human brain tissue samples (on paraffin blocks) were obtained from the Netherlands brain bank. The blocks were cut on a microtome. Paraffin-sections (8 μm) of the hippocampus-entorhinal cortex from Alzheimer’s disease brain (Braak’s stage VI) were used for immunohistochemical staining. The sections were treated with cold (+ 4 °C) 98% formic acid for 1 min followed by heat treatment in the pressure cooker (2100 Retriever) for 20 min at 121 °C. The tissue sections were incubated in blocking solution for 10 min at room temperature and then overnight with primary mouse antibody DC8E8 (1:200) and humanized AX004 (1:1000). Subsequently, the sections were incubated with a biotinylated secondary antibody (Vectastain Elite ABC Kit, Vector Laboratories) at room temperature for an hour and then reacted with avidin-biotin peroxidase-complex for 60 min (Vectastain Elite ABC Kit, Vector Laboratories), both at room temperature (25 °C). The immunoreaction was visualized with peroxidase substrate kit (Vector VIP, Vector laboratories, Ca, USA) and counterstained with methyl green (Vector Laboratories). The assessment of immunoreactivity was carried out under light microscopy at 100- to 400-fold magnification. The morphological details of tau-immunopositive lesions were defined based on the cellular localization and the pattern of staining. Digital images were taken using an Olympus BX51 microscope equipped with an Olympus DP50 digital camera (Olympus Optical Co., Ltd., Tokyo, Japan).

### FcγRs live staining

10^6^ of cells were re-suspend in 100 μL of FACS buffer (PBS with 0.5% BSA and 2 mM EDTA) and incubated with 2–5 μL antibodies: anti-CD11b, anti-CD64, anti-CD32, anti-CD16 or with respective isotype controls (see Material and Methods: Antibodies). After 20 min of incubation in the dark, the cells were washed with FACS buffer and centrifuged at 300×g for 5 min. The cell pellets were re-suspended in 400 μL of FACS buffer and analyzed by flow cytometry (BD LSRFortessa™ II cell analyzer). The IgG isotype controls were used as a negative controls for gating of CD64, CD32, CD16 and CD11b positive cells. The results are expressed as % of positive cells for individual markers.

### Tau uptake

Pure isolated microglia cells were cultivated with 50 nM tau protein labelled with Alexa Fluor488 (tau-488) and with 100 nM antibodies AX004/IgG1, AX004/IgG4, DC8E8, Rab51, or 200 nM Fab fragments of AX004/IgG1 and DC8E8, added directly into cultivation medium for 20 min at 37 °C. After incubation, cells were washed with PBS, mildly trypsinized (0.06% trypsin-EDTA) for 3 min to remove tau-antibody complex bound to the cell surface and washed with serum contained medium for residual trypsin blocking. Then the cells were collected, centrifuged and fixed for 10 min in BD FACS Lysis Solution (containing formaldehyde) at RT and analysed by flow cytometry (The BD LSRFortessa™ II cell analyzer). The unstained microglia were used as a negative control and the gate for Alexa Fluor 488-positive cells was set accordingly. The mean fluorescence intensity for AF488 was recorded as a signal from all acquired cells (positive+negative together).

To block the mouse Fcγ receptors, microglia were incubated with anti-CD16 + CD32 antibody (Abcam) and anti-CD64 (Santa Cruz Biotechnology) at a concentration of 5 μg/ml for 1 h at 37 °C prior to the tau uptake experiment.

### Immunocytochemistry

Microglia cells were plated on cover glass pre-coated with rat-tail collagen, type I (Sigma-Aldrich, St. Louis, Missouri, United States) and cultivated for 24 h in DMEM with 10% FCS. For basic analysis of microglia, cells were washed with PBS, fixed with 4% PFA-PHEM, pH 6.9 (60 mM PIPES, 25 mM HEPES, 10 mM EGTA, 2 mM MgCl_2_, PFA) for 12 min, permeabilized with 0.1% Triton X-100 in PBS (PBS-T) for 3 min followed by blocking with 5% BSA in TBS-T for 1 h. The cells were then incubated with anti-Iba1 antibody (WAKO, 1:1000) for 1 h, rinsed and incubated with secondary antibody goat anti-mouse AlexaFluor 488 (Invitrogen). For tau uptake analysis, cells were incubated with tau-488 alone and in combination with antibodies DC8E8 or Rab51 for 20 min. Then microglia were washed with PBS, mildly trypsinized (0.06% trypsin-EDTA) for 3 min and three times washed with PBS followed by fixation with 4% PFA-PHEM, pH 6.9 for 12 min. The samples were mounted in FluoroshieldTM medium with DAPI (Sigma-Aldrich). Images were captured by LSM 710 confocal microscope (Zeiss, Jena, Germany).

### Cytokine analysis

Cytokine secretion by microglia after incubation with Tau-antibody complexes was performed using Bio-Plex Pro™ Human Cytokine 27-plex assay (Bio-Rad) according to the instructions of the manufacturer. Levels of cytokines were assessed in cell culture medium from cells incubated for 20 min, 6 h or 24 h with tau or tau-antibody complexes. Cytokine levels were expressed relative to the total cellular protein measured by BCA Protein Assay (Thermo Scientific) as pg cytokines per total proteins (μg).

### Data analysis and statistics

All individual data are presented in the graphs along with means ± SD (unless stated otherwise). Microglia tau uptake fluorescence data were normalized to cells treated either with tau only or with appropriate isotype control, and then analyzed by one-way ANOVA with Tukey’s multiple comparison test. Details of data presentation and statistics used are described in figure legends.

To evaluate changes in cytokine release, data were log-transformed and changes were evaluated using repeated-measures ANOVA. At each time-point (20 min, 6 h, 24 h) changes were then evaluated using one-way ANOVA. When statistically significant, changes evoked by individual treatments at each time-point were further compared using unequal variance two-way *t* tests. All *p*-values obtained for cytokine analysis were corrected for multiple comparisons using Benjamini-Hochberg procedure to keep the false discovery rate < 0.05.

To evaluate equivalence of AX004/IgG1 and AX004/IgG4 treatments (see also Supplementary Figs. 3 and 4), we have computed 90% bootstrap confidence intervals of the difference between the means of corresponding data sets, and set the equivalence region to 40% of the range of values measured for each cytokine or microglia survival, respectively. All confidence intervals were Bonferroni-corrected for the number of comparisons in each condition.

Statistical analyses were performed using Prism software v7 (GraphPad Software, Inc.) or Matlab (The MathWorks, Inc.). A p-value < 0.05 was considered statistically significant.

## Results

### Mouse anti-tau antibody DC8E8 potentiates tau uptake into primary mouse microglia

Our previous study showed that therapeutic anti-tau antibody DC8E8 recognizes pathological tau proteins in human AD brains, discriminates between diseased and healthy tau proteins, reduces formation of insoluble oligomerized tau and mature neurofibrillary tangles in a murine tauopathy model [23]. Ex vivo studies have demonstrated that DC8E8 antibody potently blocks entry of pathological tau species into primary neurons while it does not influence viability and physiological functions of neurons [51]. In order to understand the mechanism of tau reduction at the cellular level we tested the ability of DC8E8 to target extracellular pathological oligomerized forms of tau proteins and to promote their internalization by primary mouse microglia.

We prepared heparin-induced insoluble tau oligomers generated in vitro and tagged with Alexa Fluor488 (Invitrogen, Carlsbad, California, United States). The quality of tau oligomers was verified by FTIR spectroscopy (Supplementary Fig. S1A), which confirmed a high content of beta-sheet structure reflected by a shift of amide I region maximum from 1646 cm^− 1^ (recombinant monomeric tau, disordered structure) to 1633 cm^− 1^ (heparin-polymerized tau, beta-sheet structure). Analysis of oligomerized tau by DLS and evaluated by the Dynamics software version 7.8.1.3 (Supplementary Fig. S1B) showed that sonicated oligomerized tau species have average radii of 30–90 nm with a peak at 50 nm, roughly 10-times larger than tau monomer (5 nm), corresponding to approximately 500- to 10,000-mers. The size distribution of recombinant tau polymers is comparable with brain derived tau assemblies isolated from sporadic AD, which peaked at 50–90 nm [51].

**Fig. 1:**
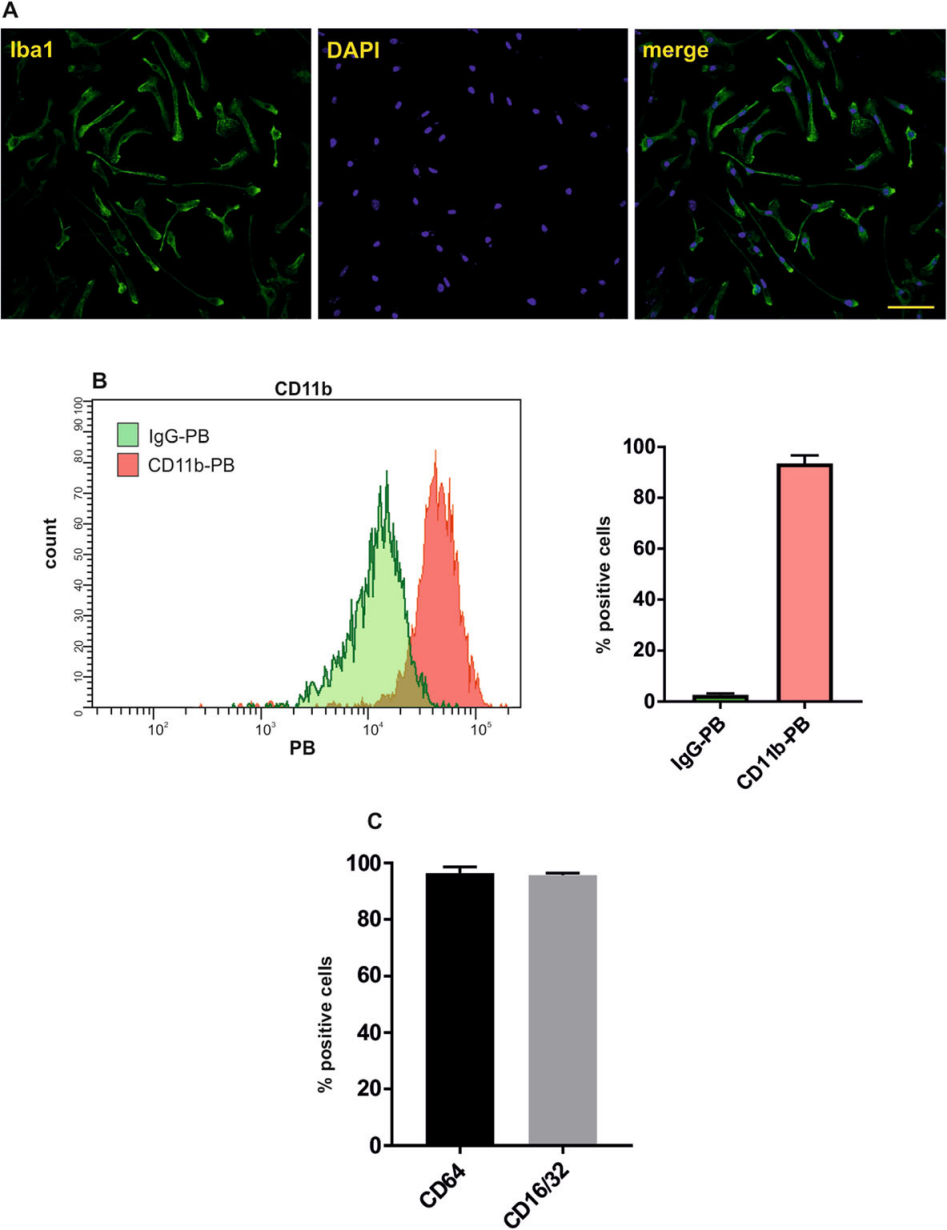
Mouse neonatal primary microglia express microglia-specific markers in culture. **a** Representative fluorescent photomicrographs showing cell morphology of cultured mouse neonatal primary microglia cells. Green, ionizing calcium-binding adaptor molecule 1 (Iba-1), and blue, DAPI nuclear staining. Scale bars, 50 μm. **b** Flow cytometry analysis confirmed purity of primary microglia culture. Histogram shows the binding for anti-CD11b compared to the binding for nonspecific IgG. Quantification of four independent microglia cultures confirmed that around 95% of cells were positive for microglia marker CD11b (mean +/− SEM). **c** Analysis of FcγRs confirmed that almost all microglia express FcγI receptor (CD64), FcγII and FcγIII receptor (CD16/32) (mean +/− SEM)

The cell morphology and microglia markers expressed by mouse neonatal microglia were characterized after 4–5 days of cultivation in vitro. The cells showed uniform resting ramified morphology with processes and elongated cell bodies (Fig. 1a). Immunostaining confirmed the presence of the specific microglia/macrophage marker, ionizing calcium-binding adaptor molecule 1 (Iba-1, Fig.1a). The purity of microglia culture, confirmed by integrin alpha M (CD11b) staining, was around 95% (Fig. 1b). All microglia expressed Fcγ receptors I (CD64), II and III (CD16/32) (Fig. 1c).

**Fig. 2:**
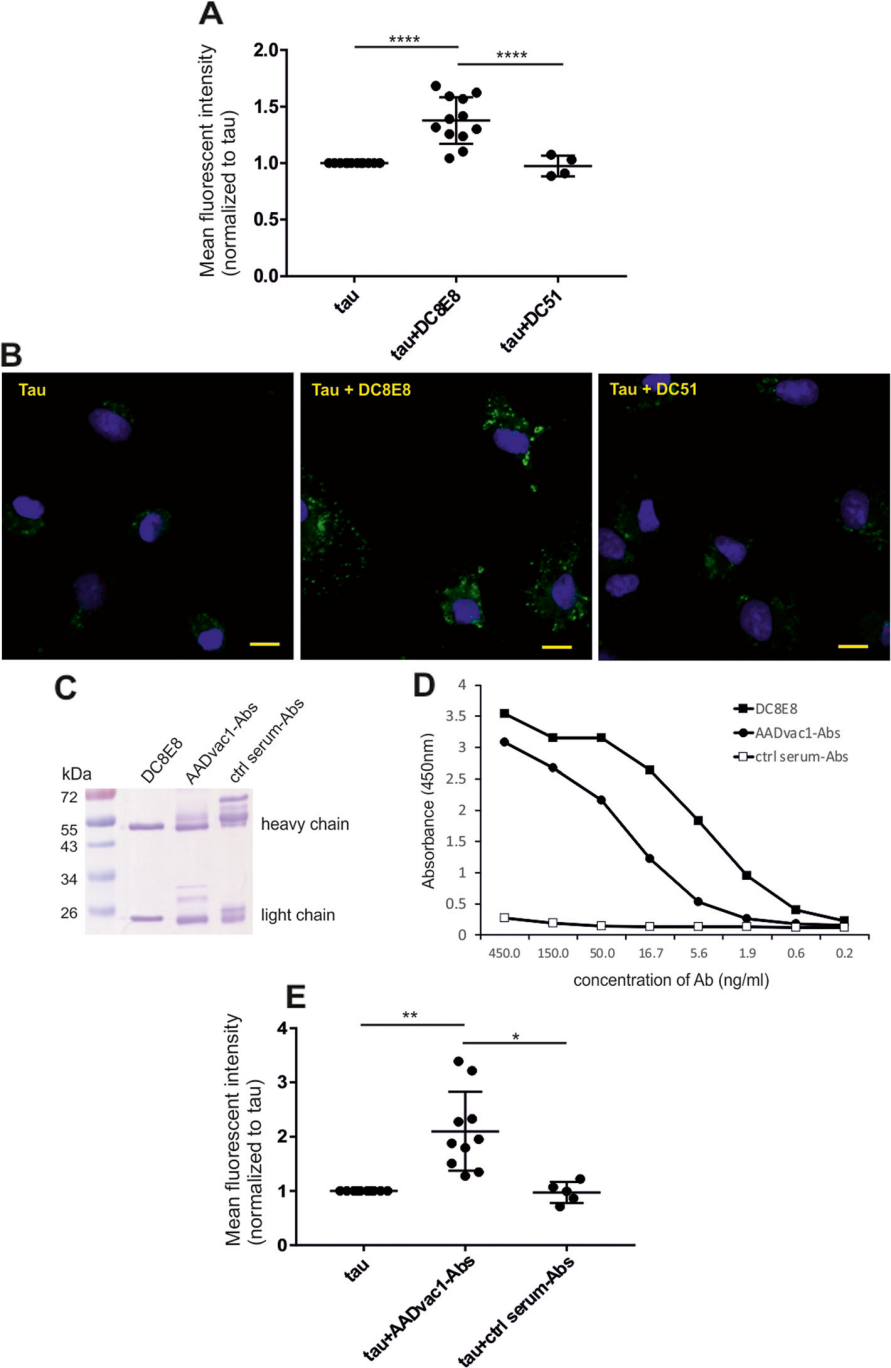
DC8E8 antibody and AADvac1-induced DC8E8-like antibodies promote tau uptake into primary mouse microglia. Increased amount of tau was detected in microglia incubated with fluorescently labelled oligomerized tau151–391/4R in the presence of DC8E8 in comparison to microglia incubated with tau alone or with tau in the presence of control monoclonal antibody DC51. **a** Tau uptake after 20 min incubation was quantified as mean fluorescence intensity using flow cytometry and normalized relative to tau only (tau vs tau+DC8E8, ****p < 0.0001, *n* = 10; tau vs tau+DC51, *p* = 0.9227, *n* = 4; tau+DC8E8 vs tau+DC51, ****p < 0.0001, n = 4), one-way ANOVA, Tukey’s multiple comparisons was used for statistical evaluation. **b** DC8E8-enhanced tau uptake by microglia was confirmed by immunostaining. Representative fluorescent photomicrographs show increased amount of Tau (green) within microglia after 20 min incubation with tau and DC8E8. Cell nuclei were stained with DAPI (blue). Scale bars, 10 μm. **c** Monoclonal profile of DC8E8 and polyclonal character of AADvac1-induced tau-specific antibodies and control sera antibodies stained with Coomasie Blue. **d** Similar tau-binding capacity of antibodies DC8E8 and AADvac1-induced tau-specific antibodies. Negative tau-binding potential of control sera antibodies was detected by sandwich enzyme-linked immunosorbent assay. **e** Serum antibodies generated after AADvac1 vaccination in mice (AADvac1-Abs) showed similar functional properties as DC8E8 antibody in primary mouse microglia. AADvac1-Abs significantly promote uptake of oligomerized tau151–391/4R into primary microglia (tau vs tau+AADvac1-Abs, **p < 0.0031, n = 10; tau vs tau+ctrl serum-Abs, *p* = 0.9998, *n* = 5; tau+AADvac1-Abs vs tau+ctrl serum-Abs, *p < 0.0154, n = 5), one-way ANOVA, Tukey’s multiple comparisons was used for statistical evaluation

The microglia were then cultivated in the presence of Alexa Fluor488-labelled oligomerized tau alone, tau with DC8E8, and tau with an irrelevant control antibody DC51 for 20 min. The evaluation of mean fluorescence intensity of all acquired cells showed that addition of anti-tau DC8E8 antibody accelerated uptake of aggregated tau by microglia (****p < 0.0001 by one-way ANOVA, Tukey’s multiple comparisons) (Fig. 2.a, b). There was no difference between tau alone and tau with the control antibody DC51 (*p* = 0.9227, Fig. 2a). Immunocytochemical staining confirmed DC8E8-enhanced tau uptake by microglia. After 20 min of incubation, we detected increased amounts of tau protein (green dots) in cellular bodies of microglia after DC8E8 mediated tau uptake (Fig. 2b).

### Serum antibodies generated after AADvac1 vaccination of mice showed similar functional properties as DC8E8 antibody in primary mouse microglia

The tau peptide corresponding to the epitope of DC8E8 was connected to a carrier and used as an active vaccine, AADvac1 that stimulates the production of DC8E8-like antibodies [22, 36, 37]. We were curious to see, whether the vaccine-induced antibodies exhibit similar phagocytosis-promoting activity like DC8E8.

Towards this end, we immunized the mice with AADvac1, isolated the induced serum anti-tau antibodies (AADvac1-Abs) and tested their ability to promote phagocytosis of oligomerized tau proteins by mouse primary microglia. Serum antibodies obtained from non-immunized mice were used as a negative control (ctrl serum-Abs). The polyclonal character of the isolated serum antibodies (AADvac1-Abs and ctrl serum-Abs) was confirmed by Coomassie brilliant blue-stained polyacrylamide gel (Fig. 2c). The binding capacity of the AADvac1-induced anti-tau antibodies to oligomerized truncated tau protein was verified by enzyme-linked immunosorbent assay (ELISA) (Fig. 2d). The difference between DC8E8 and the AADvac1-induced pool of antibodies reflects the polyclonal character of the serum antibodies further exaggerated by the detection antibody (HRP-coupled anti-mouse IgG), which might have different affinity to various mouse IgG subtypes. The antibodies from control serum did not show any binding to oligomerized tau (Fig. 2d).

Neonatal primary mouse microglia were cultivated with Alexa Fluor488-labelled oligomerized tau alone, tau+AADvac1-induced antibodies and tau+control serum antibodies for 20 min in a similar setup as described above for DC8E8 (Fig. 2ab). The evaluation of the mean fluorescence intensity of all acquired cells by flow cytometry showed that AADvac1 serum antibodies promoted tau uptake by the microglia. Polyclonal AADvac1-Abs significantly increased uptake of oligomerized tau151–391/4R into primary microglia in comparison with tau alone (tau vs tau+AADvac1-Abs, **p < 0.0031, *n* = 10; one-way ANOVA, Tukey’s multiple comparisons was used for statistical evaluation) and in comparison with tau+control serum antibodies (tau+AADvac1-Abs vs tau+ctrl serum-Abs, *p < 0.0154, *n* = 5; one-way ANOVA, Tukey’s multiple comparisons, Fig. 2e). The control serum antibodies did not promote tau uptake, the difference between tau+ctrl serum-Abs and tau alone was not significant (*p* = 0.9998, n = 5) (Fig. 2e).

Our experiments confirmed that antibodies induced by AADvac1 immunization in mice potently activate tau uptake by primary mouse microglia and are functionally similar to DC8E8.

### Humanized AX004/IgG1 potentiates tau uptake by human primary microglia more effectively than AX004/IgG4

To evaluate the activity of anti-tau antibodies in systems close to the real situation in human patients, we tested the ability of humanized forms of DC8E8 antibody, AX004/IgG1 and AX004/IgG4, to promote uptake of abnormal tau proteins by human adult primary microglia.

**Fig. 3:**
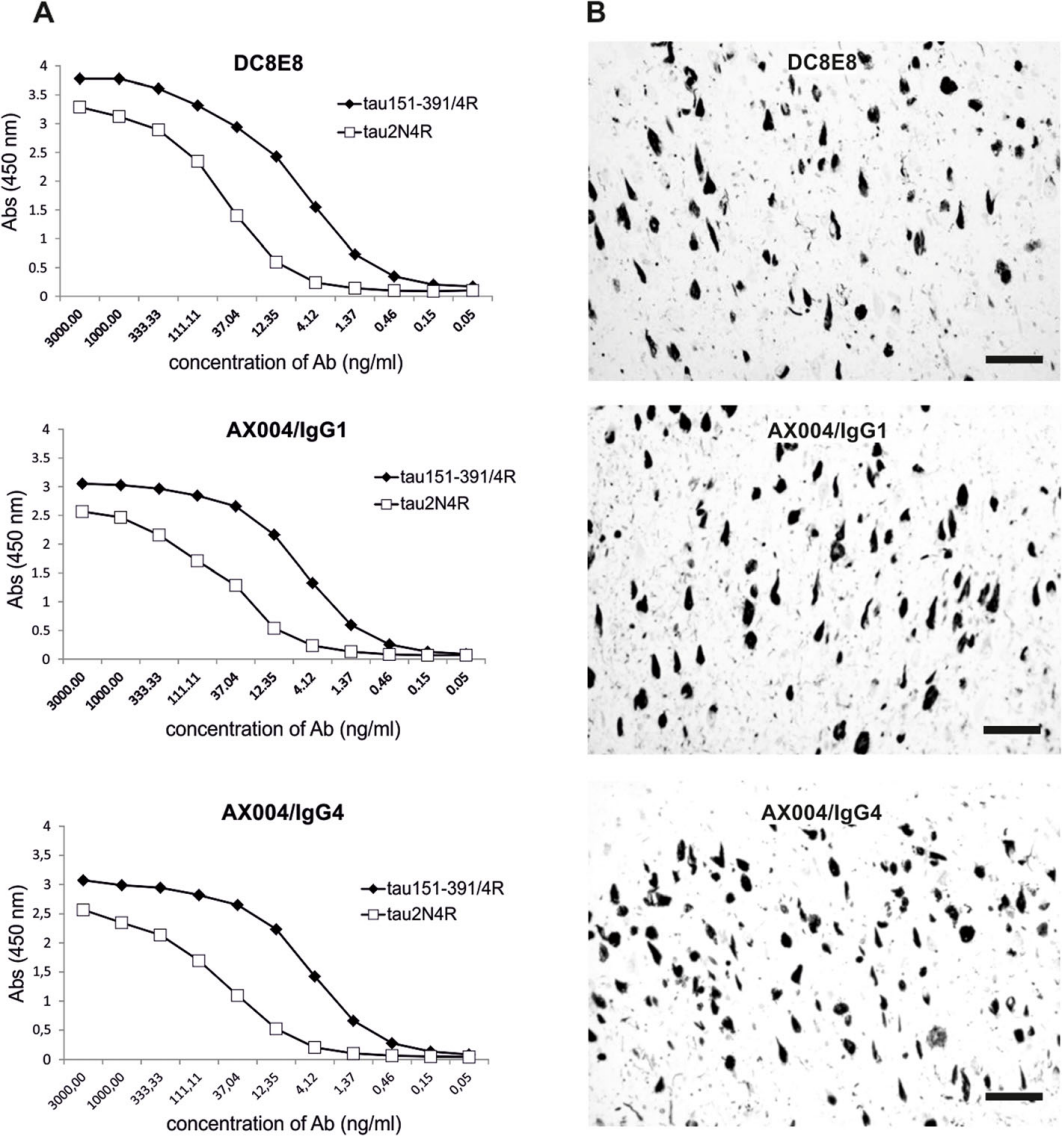
Humanized antibodies AX004/IgG1 and AX004/IgG4 and their mouse counterpart, antibody DC8E8, discriminate between pathological truncated tau protein and physiological tau. **a** Affinity of AX004/IgG1, AX004/IgG4 and DC8E8 antibodies was higher for truncated tau 151–391/4R than for the physiological tau 2N4R. The discriminatory potency of humanized AX004 (isotypes IgG1 and IgG4) is slightly higher than that of the original mouse DC8E8 antibody (EC_50_tau2N4R: AX004/IgG1 = 47.5 ng/ml, AX004/IgG4 = 53.8 ng/ml, DC8E8 = 53.5 ng/ml; EC_50_tau151–391/4R: AX004/IgG1 = 5.4 ng/ml, AX004/IgG4 = 4.7 ng/ml, DC8E8 = 7.1 ng/ml). **b** All tested antibodies AX004/IgG1, AX004/IgG4 and DC8E8 recognized a similar pattern of neurofibrillary pathology (neurofibrillary tangles and neuropil threads) at a similar level in human AD hippocampus (CA1). Scale bars, 100 μm

The tau-binding properties of humanized antibodies AX004/IgG1 and AX004/IgG4 were compared with their parental mouse antibody DC8E8. Our previous results showed that DC8E8 recognized truncated pathological tau with higher affinity than physiological full-length tau isoform 2N4R [23]. We used enzyme-linked immunosorbent assay (ELISA) to assess the immunoreactivity of humanized AX004 antibodies to tau and their ability to discriminate between pathologically truncated tau and physiological tau. The binding of all tested antibodies was higher to truncated tau151–391/4R than to the physiological tau 2N4R. The extent of discriminatory potency of humanized AX004 (both isotypes IgG1 and IgG4) was even slightly higher than that of the original mouse DC8E8 antibody, which was shown by ELISA binding curves (Fig. 3a). EC_50_ values for binding to full-length tau 2N4R were 47.5 ng/ml, 53.8 ng/ml and 53.5 ng/ml, respectively, for humanized antibody AX004/IgG1, AX004/IgG4 and DC8E8. EC_50_ values for binding to truncated tau151–391/4R were almost 10-times lower for all tested antibodies (5.4 ng/ml for AX004/IgG1, 4.7 ng/ml for AX004/IgG4 and 7.1 ng/ml for DC8E8). Importantly, isotype had no influence on immunoreactivity and the discrimination power was similar for both tested isotypes of humanized AX004 (IgG1 and IgG4). These results show that mouse DC8E8 and humanized antibody AX004 show strong preference for pathological truncated tau 151–391/4R over physiological tau 2N4R.

Immunohistochemical staining showed that humanized antibodies and mouse DC8E8 recognized similar load and the same staining pattern of neurofibrillary pathology in serial sections of human AD hippocampus (CA1) (Fig. 3b).

**Fig. 4:**
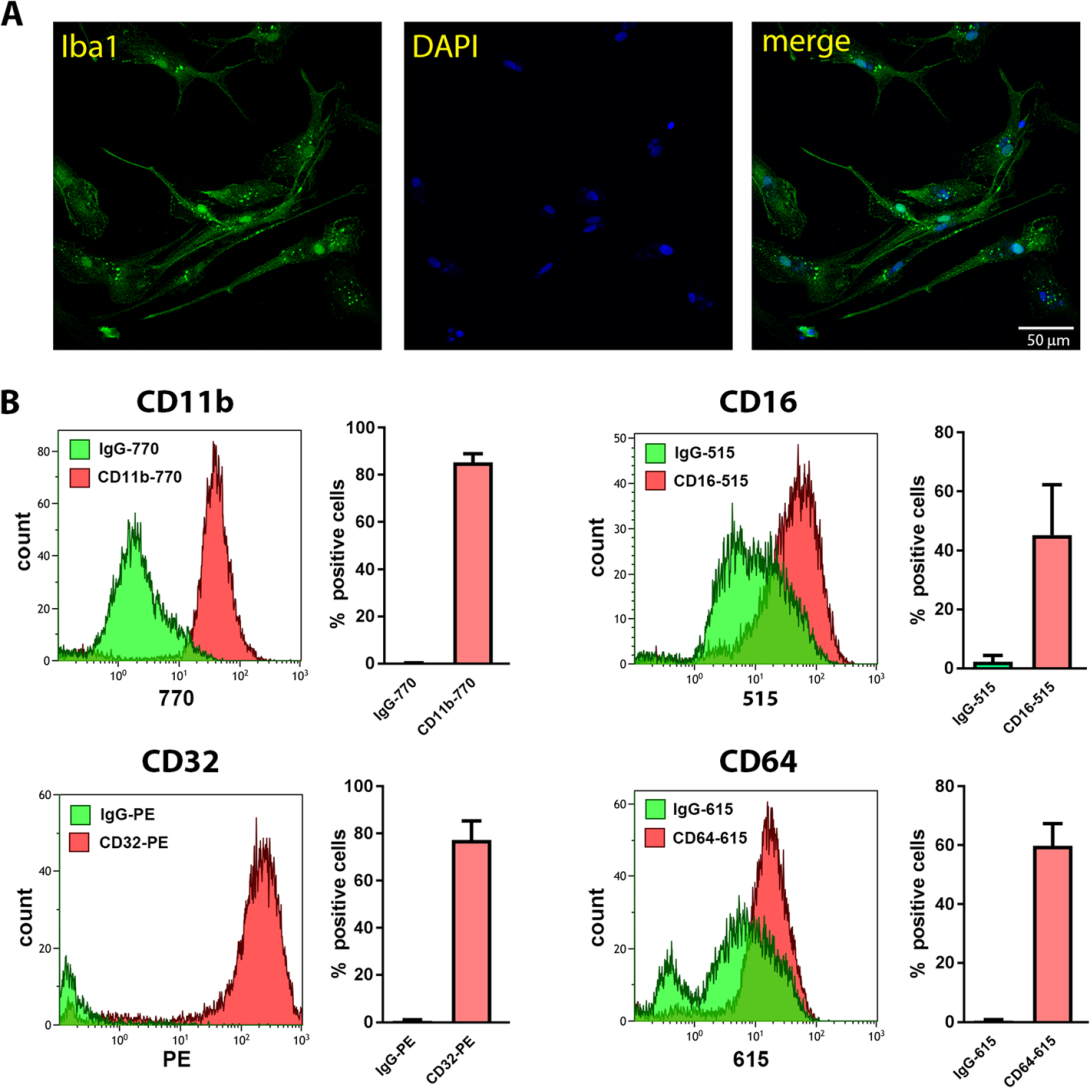
Human adult primary microglia express specific microglial markers. **a** Representative fluorescent photomicrographs of human primary microglia culture demonstrating cell morphology and purity of culture; Iba-1 (green), DAPI nuclear staining (blue). Scale bar represents 50 μm. **b** Flow cytometry analysis for CD11b, CD16, CD32 and CD64 confirmed purity of human microglia cultures. For each marker representative histograms are shown from one human microglia culture and bar graphs with percentage of positive cells from four independent microglia cultures (mean +/− SEM). Histograms and bar graphs show binding and cell positivity for anti-CD11b, −CD16, −CD32 and -CD64, respectively, compared to binding and cell positivity for nonspecific IgG

For the tau uptake assessment, human adult primary microglia were isolated post mortem from human brain tissue of 13 patients (Table 1). Basic characteristics and purity of human primary microglia cultures were evaluated similarly as described for mouse microglia. Known differences between rodent and human microglia at immune and neurological levels [45] can be further influenced by aging or neurological diseases [43, 46, 47]. Nevertheless, primary human microglia share some similarity to their rodent counterparts [45]. Our result confirmed similar cell morphology and expression of basic microglia markers Iba-1 (Fig. 4a), CD11b and all Fcγ receptors I (CD64), II (CD16) and III (CD32) in both, primary human (Fig. 4b) and mouse microglia cultures.

**Fig. 5:**
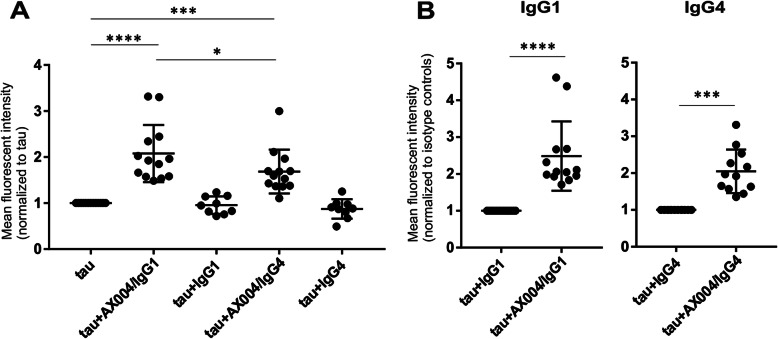
AX004/IgG1 isotype is more effective than AX004/IgG4 in potentiating tau uptake by primary human microglia cells (**a**) Flow cytometry analysis of human adult microglia cultures isolated from different donors showed increased fluorescence intensity in cells incubated with tau+AX004/IgG1 or tau+AX004/IgG4 in comparison to cells incubated with tau alone (tau vs tau+AX004/IgG1, ****p < 0.0001; tau vs tau+AX004/IgG4, ***p = 0.0003 by one-way ANOVA, Tukey’s multiple comparisons test). Comparison of the isotype IgG1 vs IgG4 of AX004 confirmed that AX004/IgG1 is more effective in facilitation tau uptake by microglia than AX004/IgG4 (*p = 0.0341, *n* = 13, by one-way ANOVA, Tukey’s multiple comparisons test). The effect of isotype controls IgG1 and IgG4 to tau uptake promotion had no effect in comparison to tau alone (tau vs tau+IgG1, *p* = 0.9988, *n* = 9; tau vs tau+IgG4, *p* = 9438, n = 9; by one-way ANOVA, Tukey’s multiple comparisons test). **b** Increased fluorescence detected after tau uptake in samples treated with AX004/IgG1 or AX004/IgG4 was significantly higher compared to cells treated with isotype controls (tau+IgG1 vs tau+AX004/IgG1, ****p < 0.0001, n = 13; tau+IgG4 vs tau+AX004/IgG4, ***p = 0.0002, n = 13 by one-way ANOVA, Tukey’s multiple comparisons test)

Human microglia were cultivated in the presence of tau alone, tau with humanized antibodies (AX004/IgG1 or AX004/IgG4) and tau with IgG1 and IgG4 isotype controls, for 20 min and evaluated by flow cytometry. Both isotypes of AX004 showed the ability to increase tau uptake by human primary microglial cells. Statistically significant increase in mean fluorescent intensity was detected in samples cultivated in the presence of tau with AX004/IgG1 or AX004/IgG4, compared with cells cultivated with tau alone (tau vs tau+AX004/IgG1, ****p < 0.0001; tau vs tau+AX004/IgG4, ***p = 0.0003 by one-way ANOVA with Tukey’s multiple comparisons test) (Fig. 5a), or compared with their isotype controls (tau+IgG1 vs tau+AX004/IgG1, ****p < 0.0001; tau+IgG4 vs tau+AX004/IgG4, ***p = 0.0002 by one-way ANOVA, Tukey’s multiple comparisons test) (Fig. 5b).

We further attempted to test whether diagnosis of donors, from which primary human microglia were derived, affected the AX004-mediated tau uptake. The cultures were separated into three groups: 4 from control cases without any neurodegeneration, 5 from tauopathy patients (AD, PSP, Pick’s disease) and 4 from patients with other neurodegeneration (MS, MSA, PD). The comparison indicated that AX004-mediated tau uptake is not affected by the patients’ diagnoses (Supplementary Fig. 2). The low number and natural heterogeneity of the samples limits the statistical power of the test and prevents us from making definitive statements about their equivalence.

Selection of an appropriate IgG isotype for a therapeutic antibody is one of the crucial steps in its development, and determines its efficacy and potential adverse events in humans. We, therefore, analysed differences between AX004/IgG1 and IgG4 isotypes in their abilities to promote uptake of abnormal tau by microglia. Antibody AX004/IgG1 showed 25% increase in tau uptake activity compared to AX004/IgG4. Statistical analysis (one-way ANOVA with Tukey’s multiple comparisons test) confirmed statistically significant differences between tau+AX004/IgG1 and tau+AX004/IgG4 (*p = 0.0341) in promoting tau uptake (Fig. 5a).

### AX004 antibody requires the Fc domain to promote tau uptake by microglia

**Fig. 6:**
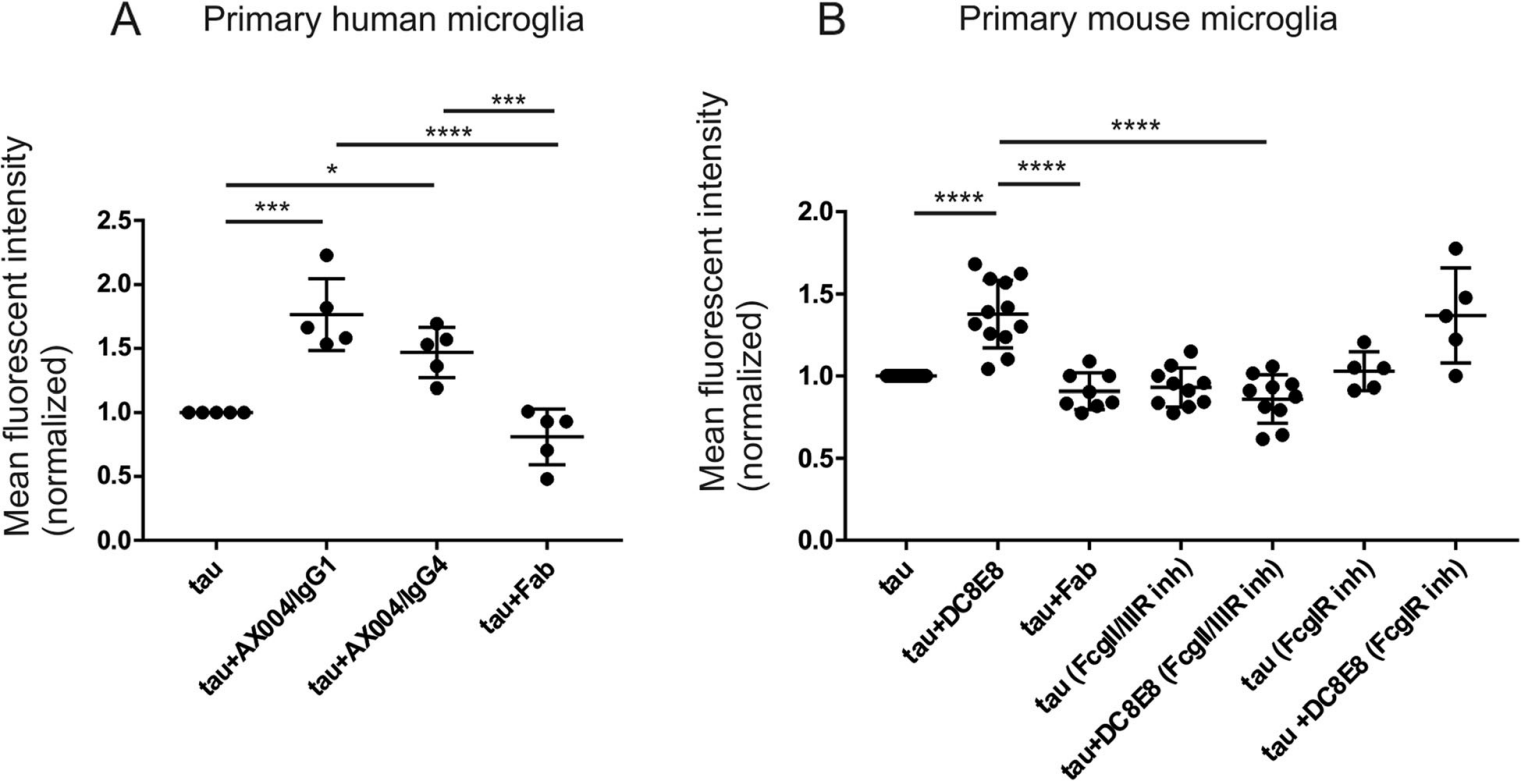
Anti-tau antibodies need the Fc domain to promote tau uptake by microglia. **a** Flow cytometry analysis of human microglia incubated with fluorescently labelled aggregated tau151–391/4R alone, in the presence tau+AX004/IgG1, or tau+AX004/IgG4, or in the presence of Fab fragment of AX004 showed that antibody-dependent tau uptake required the complete AX004 antibody. Fab fragment of AX004 did not promote uptake of tau (tau+Fab vs tau+AX004/IgG1, ****p < 0.0001, n = 5; tau+Fab vs tau+AX004/IgG4, ***p = 0.0005, n = 5 by one-way ANOVA, Tukey’s multiple comparisons test). Fluorescence intensity of tau positive cells incubated with tau together with Fab was similar to cells incubated with tau alone (tau+Fab vs tau, ns, Tukey’s multiple comparisons test). **b** Experiment with Fab fragment in mouse microglia confirmed Fc domain requirement in antibody-mediated tau uptake. Fab fragment of DC8E8 did not promote uptake of tau (tau+Fab vs tau, ns, *p* = 0.8190, *n* = 8, Tukey’s multiple comparisons test). Pre-incubation of mouse microglia with Fcγ antibodies, anti-FcγI and anti-FcγII/III 1 h before tau uptake confirmed the requirement for the Fc domain. The inhibition of FcγII/III blocked DC8E8-dependent tau uptake (****p < 0.0001, n = 10, by Tukey’s multiple comparisons test). The inhibition of FcγI receptor had no effect on DC8E8-mediated tau uptake (tau+DC8E8/FcgII/III inh vs tau+DC8E8, ns, *p* > 0.999, n = 10, by Tukey’s multiple comparisons test). For visual clarity, all values were normalized to mean fluorescence values obtained from tau-only wells

Currently, the importance of full-effector or effector-less antibodies for efficient anti-tau immunotherapy is still a matter of debate. We set out to investigate if AX004-mediated uptake required the Fc domain, which is necessary for binding of tau-antibody complex to Fcγ receptors on the microglia surface. We prepared Fab fragment of humanized AX004 and verified its tau binding properties by surface plasmon resonance. Human microglia were cultivated with fluorescently labelled oligomerized tau tau151–391/4R alone, and with tau+AX004/IgG1, tau+AX004/IgG4 or tau+Fab fragment of AX004. Flow cytometry analysis revealed that AX004 Fab fragment did not promote tau uptake while AX004 full antibodies did (tau+Fab vs tau+AX004/IgG1, ****p < 0.0001, *n* = 5; tau+Fab vs tau+AX004/IgG4, ***p = 0.0005, n = 5 by one-way ANOVA with Tukey’s multiple comparisons test). Fluorescence intensity of tau positive cells incubated with tau+Fab of AX004 and cells cultivated with tau alone were comparable (Fig. 6a).

These results show that only antibodies AX004/IgG1 or AX004/IgG4, containing both Fc and Fab domains, potentiate tau phagocytosis by human microglia. Fab fragment alone does not promote the uptake of tau. Similar results were obtained with mouse antibody DC8E8 in mouse microglia (tau+Fab vs tau, ns, *p* = 0.8190, *n* = 8, Tukey’s multiple comparisons test) (Fig. 6b).

### Antibody-dependent tau phagocytosis by microglia is mediated via FcγII/III receptors

Having determined that the Fc domain of the antibody is required for the promotion of tau uptake by microglia, we analysed which type of Fcγ receptors are engaged in antibody-mediated tau uptake. For this purpose, we tested mouse antibody DC8E8 with mouse primary microglia and used direct inhibition of individual Fcγ receptors with anti-FcγI and anti-FcγII/III antibodies. Fab fragment of DC8E8 antibody was used as a negative control. Cells were prepared and cultivated as described above, except that where appropriate the microglia were pre-incubated with anti-FcγI or anti-FcγII/III antibodies for 1 h in advance of the tau uptake experiment. Mean fluorescence intensities of tau positive cells were measured after tau uptake by flow cytometry. The inhibition of anti-FcγII/III receptors blocked DC8E8-mediated tau phagocytosis. We detected decreased tau amount in these microglia in comparison to the cells cultivated with tau+DC8E8 without the inhibition of Fcγ receptors (****p < 0.0001, *n* = 10, Tukey’s multiple comparisons test). Inhibition of FcγI receptor had no effect on DC8E8-mediated tau uptake (*p* > 0.999, n = 5, Tukey’s multiple comparisons test) (Fig. 6b).

These experiments demonstrated that antibody-dependent uptake of tau by microglia requires FcγII/III receptors.

### Immune-complexes of tau with humanized antibodies AX004/IgG1 or AX004/IgG4 do not provoke higher secretion of pro-inflammatory and anti-inflammatory cytokines by human microglia than tau alone

**Fig. 7:**
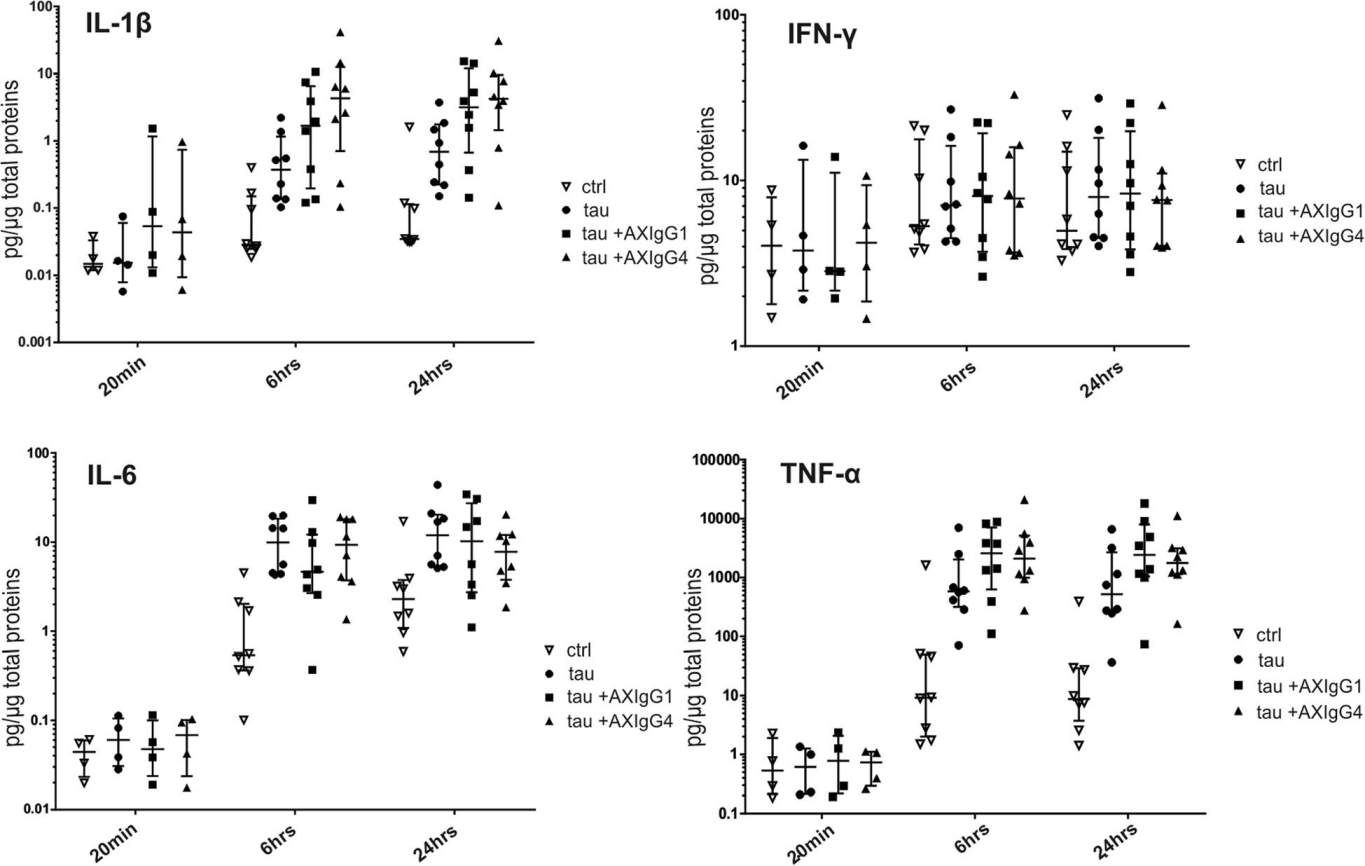
Tau+AX004/IgG1 and tau+AX004/IgG4 complexes show similar stimulation of pro-inflammatory cytokine secretion. The abilities of tau+AX004/IgG1, tau+AX004/IgG4, and tau to stimulate IL-1β, IL-6, TNF-α release were comparable, and no changes in IFN-γ were detected (see text for details). Each data point represents a different patient. All data sets are shown with their respective median values and interquartile ranges, and plotted on a logarithmic scale

**Fig. 8:**
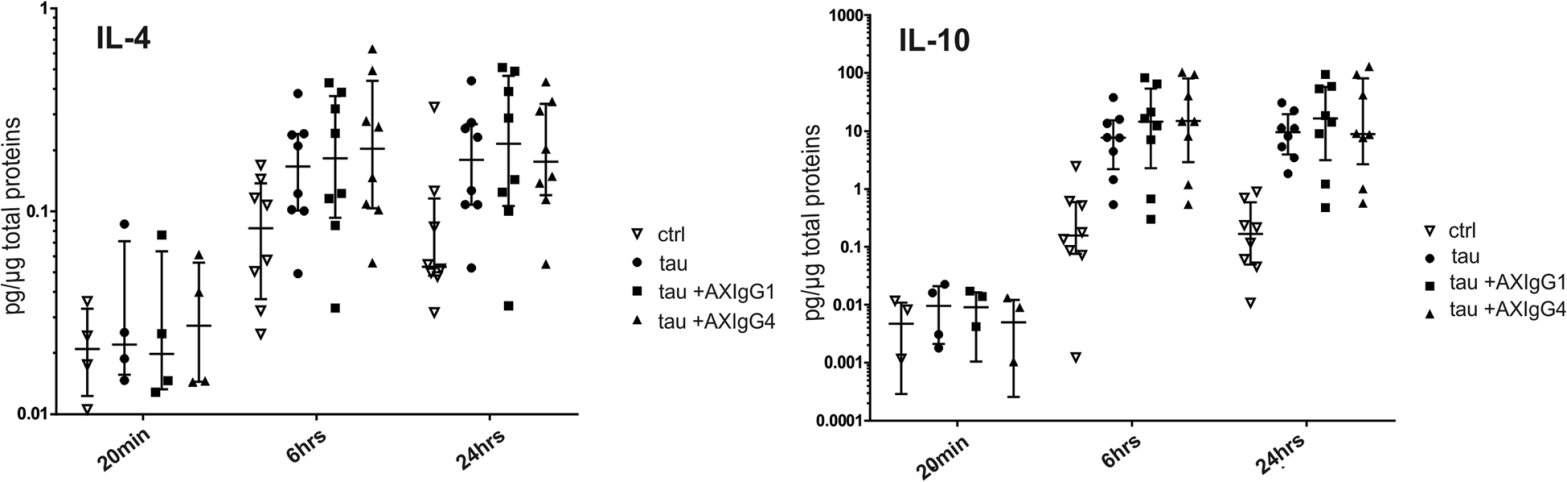
Tau+AX004/IgG1 and tau+AX004/IgG4 complexes show similar stimulation of anti-inflammatory cytokines secretion. No differences between the ability of tau+AX004/IgG1, tau+AX004/IgG4, and tau to stimulate the release of IL-4 and IL-10 were detected (see text for details). Each data point represents a different patient. All data sets are shown with their respective median values and interquartile ranges, and plotted on a logarithmic scale

FcγR engagement activates diverse downstream immunomodulatory pathways with pleiotropic functional consequences including phagocytosis of immune complexes and potential cytotoxicity caused by pro-inflammatory cytokine release [5]. We, therefore, tested whether IgG1 and IgG4 isotypes of AX004 induce release of inflammatory cytokines by human adult microglia. We incubated human microglia isolated from eight donors with tau alone as well as with tau+AX004/IgG1and tau+AX004/IgG4 immune-complexes for 20 min, 6 h and 24 h. Levels of pro-inflammatory cytokines IL-1β, IL-6, TNF-α, IFN-γ (Fig. 7) and anti-inflammatory cytokines IL-4 and IL-10 (Fig. 8) were measured in the cultivation medium using Bio-Plex Pro™ Human Cytokine 27-plex Assay. Comparison of the cytokine levels throughout the time course of the experiment revealed that incubation with tau alone increased the cytokine release over time for IL-1β, IL-6, TNF-α, IL-4 and IL-10 (*p* < 0.001) but not for IFN-γ (*p* = 0.809; repeated-measures ANOVA, Benjamini-Hochberg procedure for controlling the false discovery rate < 0.05) (Figs. 7, and 8).

We further compared the differences in the cytokine release at the individual time-points. No statistically significant changes were detected after 20 min of incubation with tau alone. After 6 and 24 h of incubation with tau alone, IL-1β, IL-6, TNF-α, and IL-10 displayed statistically significant changes in release compared to the control microglia cultures (at 6 h p = < 0.001, p < 0.001, p < 0.001, p < 0.001; at 24 h p < 0.001, *p* = 0.031, p < 0.001, p < 0.001, respectively), whereas IL-4 and IFN-γ showed no statistically significant change (at 6 h *p* = 0.111, *p* = 0.988; at 24 h p = 0.111, p = 0.988, respectively, one-way ANOVA followed by Benjamini-Hochberg procedure).

After 6 h, all treatments, i.e. incubation with tau alone, tau+AX004/IgG1, and tau+AX004/IgG4, resulted in significantly increased levels of IL-1β, IL-6, TNF-α, and IL-10 compared to the control untreated microglia cultures (IL-1β: *p* = 0.008, p = 0.03, *p* = 0.003; IL-6: *p* = 0.002, *p* = 0.02, p = 0.002; TNF-α: *p* = 0.004, *p* = 0.001, p = 0.001; IL-10: p = 0.004, p = 0.004, p = 0.004, respectively; two-way t-tests followed by Benjamini-Hochberg procedure to control the false discovery rate < 0.05). Incubation with tau+AX004/IgG4 appeared to increase IL-1β release compared to incubation with tau alone after 6 h with marginal statistical significance (*p* = 0.049).

After 24 h, incubation with tau alone, tau+AX004/IgG1, and tau+AX004/IgG4 resulted in statistically significant increase in levels of IL-1β, TNF-α and IL-10 compared to control cultures (IL-1β: p = 0.008, p = 0.001, p = 0.001; TNF-α: p = 0.001, p < 0.001, p = < 0.001; IL-10: p < 0.001, p < 0.001, p < 0.001, respectively; two-way t-tests followed by Benjamini-Hochberg procedure). Additionally, application of tau alone led to statistically significant increase in IL-6 after 24 h compared to control microglia cultures (*p* = 0.026). IL-6 levels induced by the complexes of tau+AX004/IgG1 and tau+AX004/IgG4 were at the levels of tau alone, though not statistically significantly different from the controls.

Overall, cytokine levels induced by tau+AX004 complexes were similar to those induced by tau alone at all time points (only IL-1β levels induced by tau+AX004/IgG4 complex after 6 h appeared statistically different).

In summary, tau alone stimulates the secretion of pro-inflammatory and anti-inflammatory cytokines. Immune-complexes of both tau+AX004/IgG1 and tau+AX004/IgG4 do not further increase the secretion of these cytokines (see also Supplementary Fig. 3). Furthermore, quantification of the released adenylate kinase showed that formation of the Ab-tau immune complexes does not increased cytotoxicity over tau alone (Supplementary Fig. 4).

## Discussion

In this study we demonstrated that human microglia isolated from post-mortem human brains of patients suffering from AD and other neurodegenerative disorders were able to phagocytize pathological tau from extracellular space. Their activity can be further enhanced by novel anti-tau antibodies AX004/IgG1 and AX004/IgG4, humanized versions of DC8E8 [23]. We also demonstrated that the Fc fragment of the antibody was required for promoting tau uptake by human microglia as well as mouse neonatal microglia. Both isotypes of AX004 accelerated uptake of extracellular pathological tau by human microglia regardless of patients’ diagnoses. In addition, the IgG1 isotype of AX004 antibody was more effective in the promoting tau uptake than its corresponding IgG4 isotype, consistent with the generally accepted less efficient effector function of IgG4 subclass [12].

Over the past ten years, immense effort has been invested in the development of immunotherapies targeting the pathological tau proteins in AD. It is generally expected that therapeutic anti-tau antibodies will bind abnormal tau proteins, prevent their internalization by neurons and promote their removal by microglia, the brain resident macrophages. Effective tau immunotherapy, which increases microglial appetite for abnormal tau, might reverse the pathogenic cascade. The molecular mechanism of tau removal was usually tested in in vitro systems, mostly using primary neonatal mouse microglia or stable microglia-like cell lines [2, 3, 15, 27, 31, 33]. Recently, cultivation of human primary microglia have also been established [7, 13, 17, 34, 35] and important phenotypical differences between human primary microglia and other microglia cell models were described [34, 55]. The differences between human and mouse models were further emphasized by detailed analysis of aged and disease-related primary human microglia [14, 16, 38]. In addition, in light of immunotherapy development the use of a human preclinical model is essential since human antibodies specifically interact with human Fc receptors.

Thus, these findings significantly underline the importance of primary human microglia in preclinical testing of potential human therapeutics that engage microglia. Herewith human primary microglia bridge the gap between cell models from non-human species to the human brain and lend more credence to the interpretation of the results.

We isolated adult primary human microglia from the brains of diseased patients suffering from several neurodegenerative disorders (AD, PD, FTD, DLB, PSP, MS and MSA). These cells were exposed to genuine ailing human CNS microenvironment. All human microglia cultures expressed basic microglia markers such as Iba-1, CD11b, Fcγ receptors: I (CD64), II (CD16) and III (CD32), showed the potential to become activated, were able to phagocytose truncated oligomerized tau proteins and stimulated immune response. The tau uptake appetite was further promoted by anti-tau antibodies AX004/IgG1 and AX004/IgG4, whereas the Fab fragment of AX004 did not promote this activity. These data confirm that human antibody-mediated tau phagocytosis by human microglia is Fc-dependent, similar to what we found on DC8E8 dependence on FcγRII/III receptors. This is in concordance with several independent studies performed with various mouse anti-tau antibodies on mouse microglia [2, 3, 15, 27, 31].

In context with the clinical trial on the first-in-man active tau vaccine AADvac1, designed based on the DC8E8 epitope [22, 36, 37], we were interested whether the antibodies elicited after AADvac1 vaccination exhibit similar tau-uptake promoting properties. Similarly to our previous preclinical study on transgenic rats expressing truncated tau, we showed that AADvac1 vaccine induced primarily IgG antibody isotypes with preference for IgG1, and only very low levels of IgM antibodies [23]. AADvac1-induced serum antibodies showed very similar phagocytosis-promoting properties to the monoclonal DC8E8 antibody. This finding confirms that the active vaccine AADvac1 targets abnormal forms of tau, promotes their elimination and has disease modifying potential.

Based on the observations from amyloid immunotherapy, anti-tau antibodies with effector function could be more effective in clearing pathological tau by microglia than antibodies without the effector function. The effect of tau antibodies on tau microglial uptake is an important feature of their therapeutic mode of action and should be taken into account in the pre-clinical efficacy studies. Fc-FcγR interactions represent the key component of in vivo activity of all therapeutic antibodies and initiate a number of immunomodulatory functions. These include cellular activation, phagocytosis and pro-inflammatory cytokine release [6, 52].

It has recently been shown that an anti-tau antibody with full effector function in the presence of tau oligomers increased production of IL-1ß, IL-6 and TNF-α by primary microglia, which led to MAP 2 fragmentation in co-cultured primary neuronal cells, while an antibody without its effector function did not cause the MAP 2 fragmentation. The authors concluded that antibodies with effector function may have deleterious effect on neuronal cells [27]. However, further supporting data on neuronal toxicity driven by activated microglia are missing (impaired viability or nuclear morphology, ATP levels etc.).

In our study, we tested the tau-antibody immune-complexes (tau+AX004/IgG1 and tau+AX004/IgG4) for their ability to stimulate the immune response in human microglia cultures. Even though both immune-complexes stimulated release of pro-inflammatory cytokines (IL-1β, IL-6, TNF-α, IFN-γ) between 20 min and 24 h, the levels of cytokines were very similar for both antibody isotypes. Furthermore, the increase in cytokine levels was apparently activated by tau alone and the presence of antibodies in the cultivation media did not contribute to additional enhancement of the cytokine release. This is consistent with our previous study where we showed that recombinant truncated tau protein alone induced transformation of mouse primary microglia into the reactive phenotype accompanied by a release of nitric oxide, proinflammatory cytokines (IL-1β, IL-6, TNF-α), and tissue inhibitor of metalloproteinase-1 [24]. Besides, both tau+AX004/IgG1 and tau+AX004/IgG4 complexes also induced increased expression of anti-inflammatory cytokines (IL-4 and IL-10). Cytokines IL-4 and IL-10 suppress inflammation through the inhibition of IL-1β, IL-6, TNF-α secretion by microglia (for review see [54]). This indicated that increased levels of anti-inflammatory cytokines IL-4 and IL-10 in human microglia culture could result in local autocrine/paracrine regulation of pro-inflammatory actions and could lead to a balanced activity promoting phagocytosis. Several studies on microglia showed competition of pro-inflammatory and anti-inflammatory stimuli, where addition of IL-4 or IL-10 to activated microglia decreased induction of other pro-inflammatory cytokines [21, 26, 30, 48].

Currently, the IgG4 isotype prevails in the passive tau immunotherapy clinical trials [36], with the aim to avoid undesirable activation within the central nervous system. However, clinical studies on amyloid immunotherapy showed that anti-amyloid antibodies with IgG1 backbone were more effective in clearing amyloid pathology from human brains than those with IgG4. In the PRIME study, aducanumab (epitope: N-terminus Aβ3–6, IgG1 isotype) reduced brain Aβ plaques as measured by amyloid PET imaging in a dose- and time-dependent fashion [42]. Preclinical studies revealed that aducanumab enhanced recruitment of microglia to amyloid plaques via engagement of Fcγ receptors. In a recent study on bapineuzumab (epitope: N-terminus Aβ1–5, IgG1 isotype) the authors demonstrated that Alzheimer’s Related Imaging Abnormality-Edema (ARIA-E) was associated with larger reductions in amyloid PET, suggesting that in treated cases ARIA-E might be related to increased Aβ efflux from the brain [29]. In addition, higher dose of gantenerumab (epitope: N-terminus Aβ1–10 and central region Aβ18–27, IgG1 isotype) reduced amyloid load as measured by amyloid PET, which was indicative of successful target engagement in the brain [39]. On the other hand, crenezumab (epitope: N-terminus pyroglutamate - Aβ1–15), an anti-amyloid humanized antibody with IgG4 backbone, did not exhibit significant reduction of amyloid pathology on PET imaging [40]. In an in vitro study, crenezumab was significantly less effective in stimulation the uptake of Aβ oligomers by microglia than its IgG1 counterpart [1]. Although extrapolation of results from Aβ immunotherapy could be speculative, the mechanism of removal of extracellular tau may show similarities with the clearance of extracellular Aβ. Previous findings suggest, that the effector function of an antibody plays a crucial role in the antibody-mediated uptake of Aβ. The same appears to hold for tau active immunotherapy. In line with results from clinical and preclinical Aβ immunotherapy studies, our data indicates that IgG1 antibodies work more effective in anti-tau immunotherapy. Vaccination with AADvac1 in the Phase 1 clinical trial generated predominantly IgG1 antibody response in AD patients [37]. So far, no safety signals have been observed throughout the course of the AADvac1 Phase 1 and Phase 2 clinical trials, further supporting the notion that IgG1 isotype should be preferred for clinical development. These data indicate that the issue of possible neurotoxicity of tau IgG1 antibodies should be reassessed in the light of new results coming from the ongoing clinical trials. Since the IgG1 isotype was more effective in facilitating the uptake of extracellular abnormal tau by adult human microglia than the IgG4 isotype, AX004/IgG1 is more promising for immunotherapy. In combination with its ability to block spreading of tau pathology [51] AX004 is a functional therapeutic candidate for treatment of neurofibrillary pathology.

## Supplementary information

**Additional file 1. Supplementary Fig. S1 Characterization of heparin-induced tau oligomers.** (A) FTIR spectroscopy of tau confirmed a structural change after in vitro fibrillization of human truncated tau151–391/4R. The prevalent disorder of monomeric tau (dashed line) changed to beta-rich structure (solid line). (B) Dynamic light scattering measurements showed high-molecular weight tau species in heparin-aggregated recombinant tau151–391/4R with an average radius spanning from 30 to 90 nm (solid line). Monomeric tau151–391/4R is included for comparison (dashed line).

**Additional file 2. Supplementary Fig. S. 2 AX004-mediated tau uptake is not affected by diagnosis of donors from which primary human microglia cultures were derived.** Human microglia cultures from 4 control cases without neurodegeneration, 5 tauopathy patients (AD, PSP, Pick’s disease) and 4 patients with other neurodegeneration (MS, MSA, PD) were compared for tau uptake. Diagnosis of patients did not have an effect on antibody-mediated tau uptake by microglia. The values were normalized to the corresponding isotype control values.

**Additional file 3. Supplementary Fig. S. 3 Tau + AX004/IgG1 and Tau + AX004/IgG4 complexes show equivalent stimulation of anti-inflammatory cytokines secretion.** The equivalence Tau+AX004/IgG1 and Tau+AX004/IgG4 complexes in stimulating secretion of anti-inflammatory cytokines was evaluated by computing 90% bootstrap confidence intervals of the difference between the means of the corresponding data sets. The confidence intervals were Bonferroni-corrected and compared with equivalence regions defined as +/− 40% of the range of values for each cytokine. In each panel, horizontal lines show the confidence intervals of differences between means (black circles), solid vertical lines show no-difference, and dashed vertical lines show the edges of equivalence regions. The equivalence regions for each cytokine were set as follows (in pg/μg): IL-1β +/− 16.39; IL-6 17.52; TNF-α 8302; IL-4 0.248; IL-10 51.28; IFN-γ 12.61.

**Additional file 4. Supplementary Fig. S. 4 The tau + antibody immune-complexes did not show higher toxicity in human primary microglia cultures compared to tau alone.** The ToxiLight™ bioassay kit (Lonza) was used for detection of the release of adenylate kinase (AK) from damaged cells. Cell culture medium from untreated microglia, microglia treated with tau alone as well as with tau+AX004/IgG1 and tau+AX004/IgG4 immune-complexes for 6 h and 24 h were used for analysis. The result did not show a statistically significant difference between cytotoxicity induced by tau+antibody immune-complexes and tau alone (6 h: tau vs tau+AX004/IgG1, *p* = 0.7968; tau vs tau+AX004/IgG4, *p* = 0.8234; 24 h: tau vs tau+AX004/IgG1, *p* = 0.3920; tau vs tau+AX004/IgG4, *p* = 0.8210; *n* = 8, by one-way ANOVA, Tukey’s multiple comparisons test, panel A). Similarly, we did not detect statistically significant difference between tau+AX004/IgG1 and tau+AX004/IgG4 immune-complexes (A). For test of equivalence of the two treatments on microglial survival we computed Bonferroni-corrected bootstrap confidence intervals and compared them with pre-set equivalence regions (B). This analysis shows that the impact of tau+AX004/IgG1 and tau+AX004/IgG4 treatments on microglial survival can be considered equivalent.
