# Supplementary figures and images for: Humanized tau antibodies promote tau uptake by human microglia without any increase of inflammation
Source: Acta Neuropathol Commun. 2020 May 29;8:74. doi: 10.1186/s40478-020-00948-z (PMC7257136; doi:10.1186/s40478-020-00948-z)

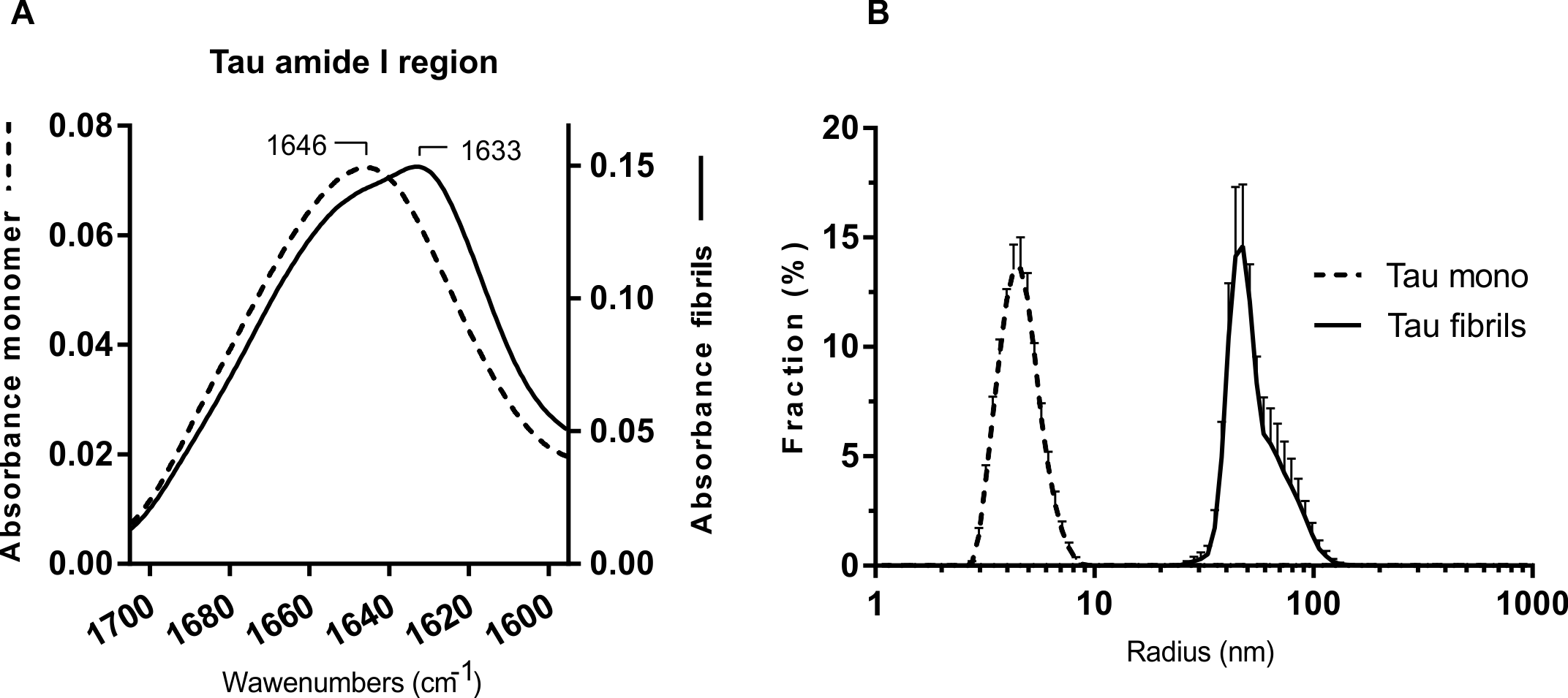

Supplement: Supplementary file 1 — Additional file 1. Supplementary Fig. S1 Characterization of heparin-induced tau oligomers. (A) FTIR spectroscopy of tau confirmed a structural change after in vitro fibrillization of human truncated tau151–391/4R. The prevalent disorder of monomeric tau (dashed line) changed to beta-rich structure (solid line). (B) Dynamic light scattering measurements showed high-molecular weight tau species in heparin-aggregated recombinant tau151–391/4R with an average radius spanning from 30 to 90 nm (solid line). Monomeric tau151–391/4R is included for comparison (dashed line). [file 40478_2020_948_MOESM1_ESM.tif]

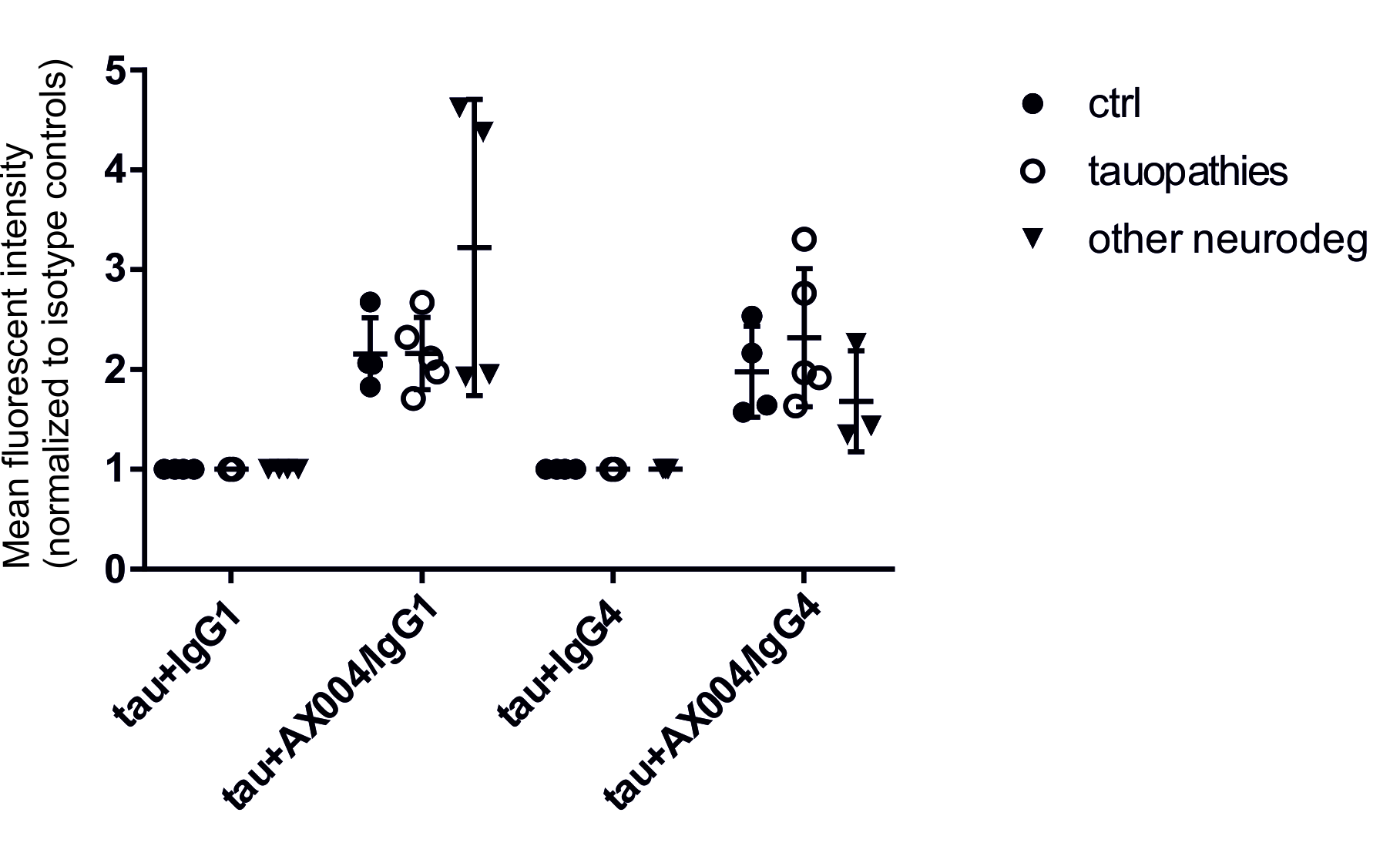

Supplement: Supplementary file 2 — Additional file 2. Supplementary Fig. S. 2 AX004-mediated tau uptake is not affected by diagnosis of donors from which primary human microglia cultures were derived. Human microglia cultures from 4 control cases without neurodegeneration, 5 tauopathy patients (AD, PSP, Pick’s disease) and 4 patients with other neurodegeneration (MS, MSA, PD) were compared for tau uptake. Diagnosis of patients did not have an effect on antibody-mediated tau uptake by microglia. The values were normalized to the corresponding isotype control values. [file 40478_2020_948_MOESM2_ESM.tif]

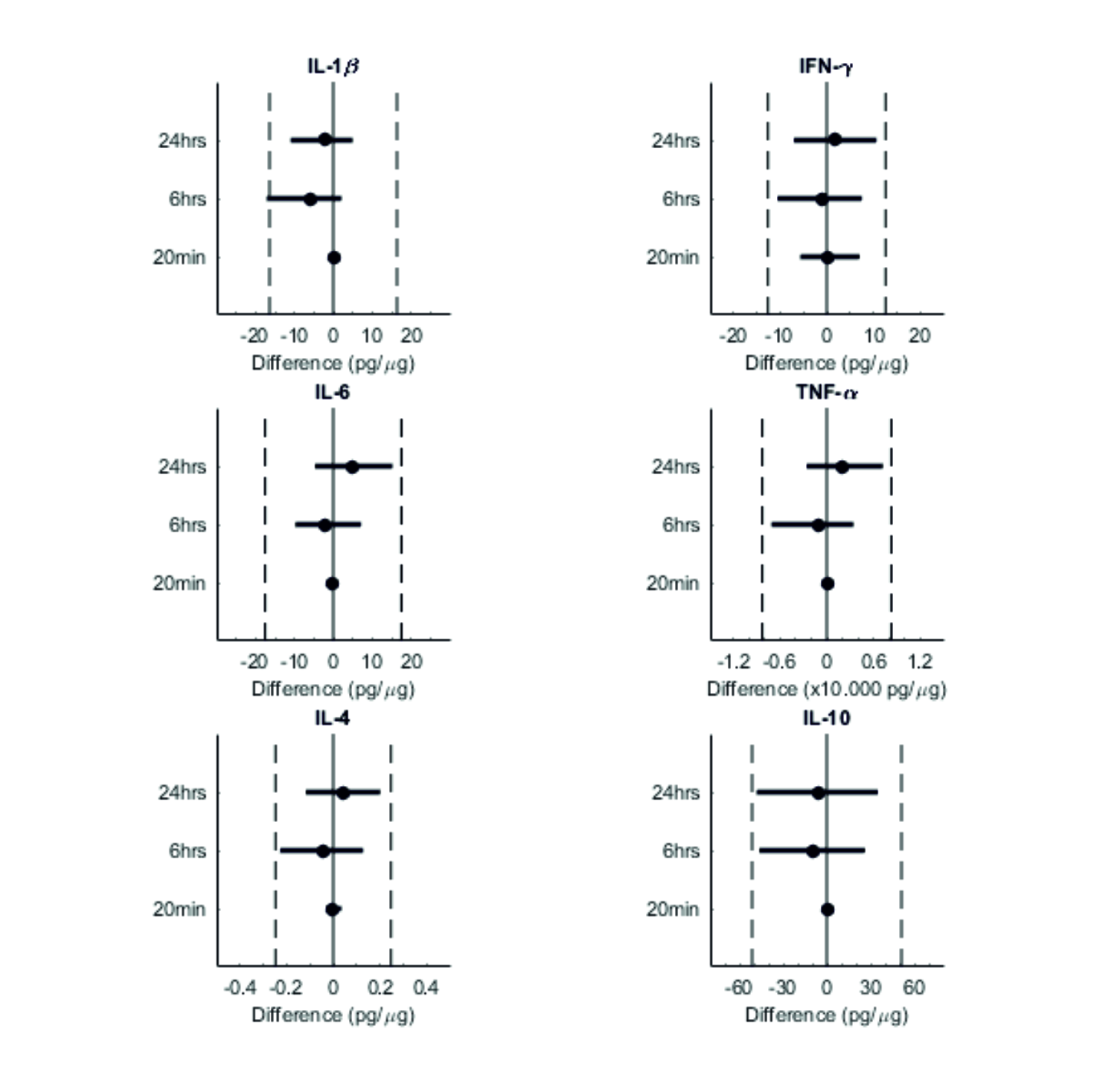

Supplement: Supplementary file 3 — Additional file 3. Supplementary Fig. S. 3 Tau + AX004/IgG1 and Tau + AX004/IgG4 complexes show equivalent stimulation of anti-inflammatory cytokines secretion. The equivalence Tau+AX004/IgG1 and Tau+AX004/IgG4 complexes in stimulating secretion of anti-inflammatory cytokines was evaluated by computing 90% bootstrap confidence intervals of the difference between the means of the corresponding data sets. The confidence intervals were Bonferroni-corrected and compared with equivalence regions defined as +/− 40% of the range of values for each cytokine. In each panel, horizontal lines show the confidence intervals of differences between means (black circles), solid vertical lines show no-difference, and dashed vertical lines show the edges of equivalence regions. The equivalence regions for each cytokine were set as follows (in pg/μg): IL-1β +/− 16.39; IL-6 17.52; TNF-α 8302; IL-4 0.248; IL-10 51.28; IFN-γ 12.61. [file 40478_2020_948_MOESM3_ESM.tif]

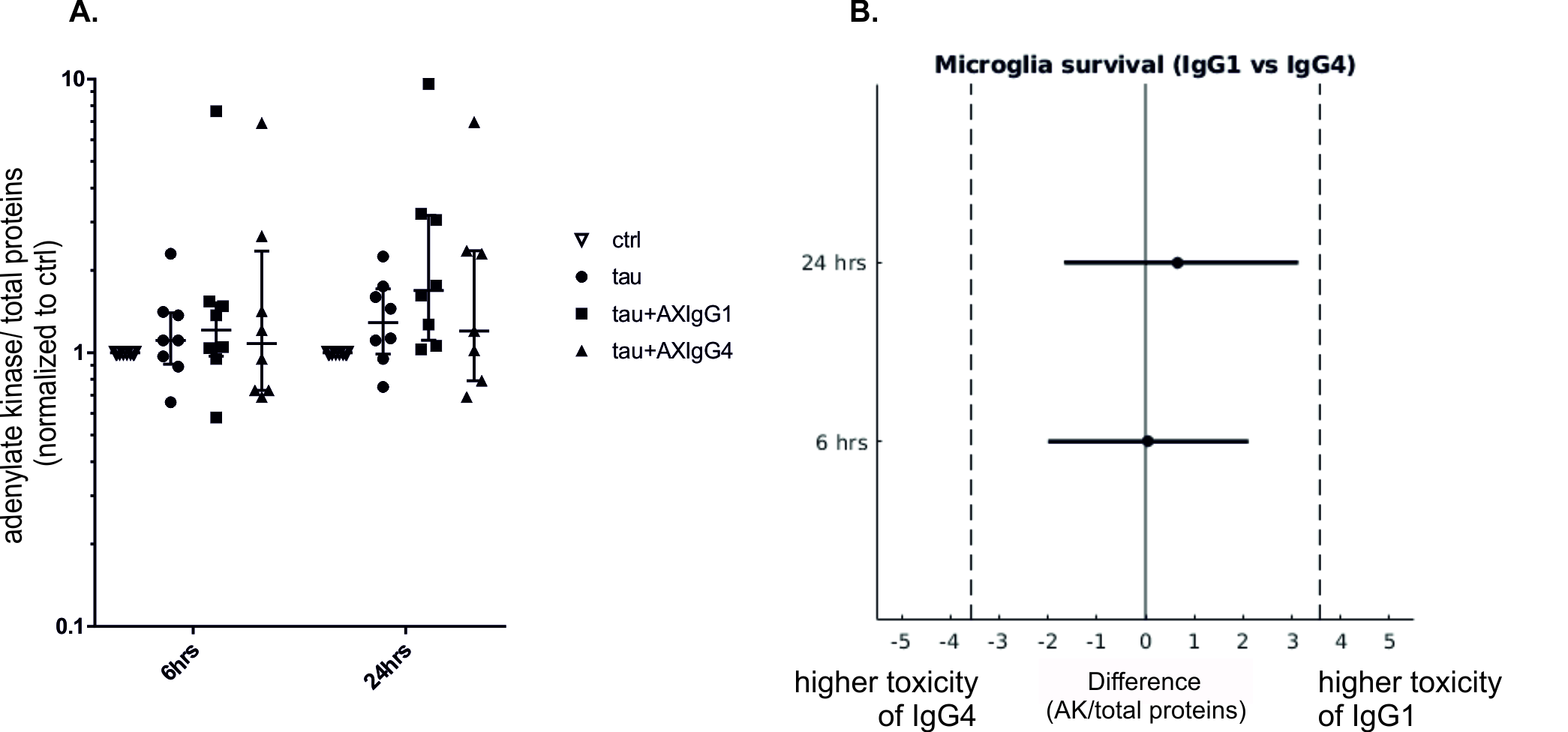

Supplement: Supplementary file 4 — Additional file 4. Supplementary Fig. S. 4 The tau + antibody immune-complexes did not show higher toxicity in human primary microglia cultures compared to tau alone. The ToxiLight™ bioassay kit (Lonza) was used for detection of the release of adenylate kinase (AK) from damaged cells. Cell culture medium from untreated microglia, microglia treated with tau alone as well as with tau+AX004/IgG1 and tau+AX004/IgG4 immune-complexes for 6 h and 24 h were used for analysis. The result did not show a statistically significant difference between cytotoxicity induced by tau+antibody immune-complexes and tau alone (6 h: tau vs tau+AX004/IgG1, p = 0.7968; tau vs tau+AX004/IgG4, p = 0.8234; 24 h: tau vs tau+AX004/IgG1, p = 0.3920; tau vs tau+AX004/IgG4, p = 0.8210; n = 8, by one-way ANOVA, Tukey’s multiple comparisons test, panel A). Similarly, we did not detect statistically significant difference between tau+AX004/IgG1 and tau+AX004/IgG4 immune-complexes (A). For test of equivalence of the two treatments on microglial survival we computed Bonferroni-corrected bootstrap confidence intervals and compared them with pre-set equivalence regions (B). This analysis shows that the impact of tau+AX004/IgG1 and tau+AX004/IgG4 treatments on microglial survival can be considered equivalent. [file 40478_2020_948_MOESM4_ESM.tif]
